# Selective Targeting of Cancerous Mitochondria and Suppression of Tumor Growth Using Redox-Active Treatment Adjuvant

**DOI:** 10.1155/2020/6212935

**Published:** 2020-11-02

**Authors:** Rumiana Bakalova, Severina Semkova, Donika Ivanova, Zhivko Zhelev, Thomas Miller, Tsuguhide Takeshima, Sayaka Shibata, Dessislava Lazarova, Ichio Aoki, Tatsuya Higashi

**Affiliations:** ^1^Quantum-State Controlled MRI Group, Institute for Quantum Life Science, National Institutes for Quantum and Radiological Science and Technology (QST), 4-9-1 Anagawa, Chiba 263-8555, Japan; ^2^Functional and Molecular Imaging Team, Department of Molecular Imaging and Theranostics, National Institute of Radiological Sciences (NIRS), QST, 4-9-1 Anagawa, Chiba 263-8555, Japan; ^3^Medical Faculty, Sofia University, 1 Koziak Str., Sofia 1407, Bulgaria; ^4^Institute of Biophysics and Biomedical Engineering, Bulgarian Academy of Sciences, 21 Acad. G. Bonchev Str., Sofia 1113, Bulgaria; ^5^Faculty of Veterinary Medicine, Trakia University, 11 Armejska Str., Stara Zagora 6000, Bulgaria; ^6^Medical Faculty, Trakia University, 11 Armejska Str., Stara Zagora 6000, Bulgaria; ^7^IC-MedTech Co., San Diego, CA, USA; ^8^Radiation and Cancer Biology Group, Department of Charged Particle Therapy Research, National Institute of Radiological Sciences (NIRS), QST, 4-9-1 Anagawa, Chiba 263-8555, Japan

## Abstract

Redox-active substances and their combinations, such as of quinone/ascorbate and in particular menadione/ascorbate (M/A; also named Apatone®), attract attention with their unusual ability to kill cancer cells without affecting the viability of normal cells as well as with the synergistic anticancer effect of both molecules. So far, the primary mechanism of M/A-mediated anticancer effects has not been linked to the mitochondria. The aim of our study was to clarify whether this “combination drug” affects mitochondrial functionality specifically in cancer cells. Studies were conducted on cancer cells (Jurkat, Colon26, and MCF7) and normal cells (normal lymphocytes, FHC, and MCF10A), treated with different concentrations of menadione, ascorbate, and/or their combination (2/200, 3/300, 5/500, 10/1000, and 20/2000 *μ*M/*μ*M of M/A). M/A exhibited highly specific and synergistic suppression on cancer cell growth but without adversely affecting the viability of normal cells at pharmacologically attainable concentrations. In M/A-treated cancer cells, the cytostatic/cytotoxic effect is accompanied by (i) extremely high production of mitochondrial superoxide (up to 15-fold over the control level), (ii) a significant decrease of mitochondrial membrane potential, (iii) a decrease of the steady-state levels of ATP, succinate, NADH, and NAD^+^, and (iv) a decreased expression of programed cell death ligand 1 (PD-L1)—one of the major immune checkpoints. These effects were dose dependent. The inhibition of NQO1 by dicoumarol increased mitochondrial superoxide and sensitized cancer cells to M/A. In normal cells, M/A induced relatively low and dose-independent increase of mitochondrial superoxide and mild oxidative stress, which seems to be well tolerated. These data suggest that all anticancer effects of M/A result from a specific mechanism, tightly connected to the mitochondria of cancer cells. At low/tolerable doses of M/A (1/100-3/300 *μ*M/*μ*M) attainable in cancer by oral and parenteral administration, M/A sensitized cancer cells to conventional anticancer drugs, exhibiting synergistic or additive cytotoxicity accompanied by impressive induction of apoptosis. Combinations of M/A with 13 anticancer drugs were investigated (ABT-737, barasertib, bleomycin, BEZ-235, bortezomib, cisplatin, everolimus, lomustine, lonafarnib, MG-132, MLN-2238, palbociclib, and PI-103). Low/tolerable doses of M/A did not induce irreversible cytotoxicity in cancer cells but did cause irreversible metabolic changes, including: (i) a decrease of succinate and NADH, (ii) depolarization of the mitochondrial membrane, and (iii) overproduction of superoxide in the mitochondria of cancer cells only. In addition, M/A suppressed tumor growth in vivo after oral administration in mice with melanoma and the drug downregulated PD-L1 in melanoma cells. Experimental data suggest a great potential for beneficial anticancer effects of M/A through increasing the sensitivity of cancer cells to conventional anticancer therapy, as well as to the immune system, while sparing normal cells. We hypothesize that M/A-mediated anticancer effects are triggered by redox cycling of both substances, specifically within dysfunctional mitochondria. M/A may also have a beneficial effect on the immune system, making cancer cells “visible” and more vulnerable to the native immune response.

## 1. Introduction

The scientific community and the pharmaceutical industry have invested enormous efforts and resources in the discovery and development of drugs with the goal of targeting cancerous mitochondria. This goal dates from the middle of the last century, when Warburg's studies suggested that the mitochondria are dysfunctional in cancer. Currently, this is considered a hallmark of carcinogenesis and used as a molecular platform for the development of “targeted” anticancer therapy. The nature of mitochondrial dysfunction in cancer has not yet been discovered. There are many examples of molecules acting at the mitochondrial level. However, there are serious debates and doubts about whether they are selective for cancerous mitochondria or they attack the mitochondria of normal cells too.

It is well known that the mitochondria are crucial for the redox regulation and signaling, as well as for cellular longevity. The regulation of mitochondrial homeostasis and their integrity and autophagy determines the choice of the cell—“to be or not to be” (to live or die). There is a great interest in the *discovery and development of redox-active substances to target the mitochondria and modulate their integrity and function*. However, there is no significant progress in the development of redox modulators that can selectively distinguish and attack the dysfunctional mitochondria. This is a challenge for scientists and pharmaceutics, especially in the field of anticancer therapy.

The redox-active combinations of quinone/ascorbate, particularly of menadione/ascorbate (M/A; also termed Apatone®, the ratio of 1/100 mol/mol menadione to ascorbate), has attracted the attention of researchers for more than 20 years with the unusual ability of the provitamin/vitamin combination to kill cancer cells without affecting the viability of normal cells, as well as with the synergistic anticancer and antifibrotic effects of both molecules [1–10]. Recently, this unique combination has received Orphan Drug Designation for treating rare diseases and conditions including (i) metastatic or locally advanced inoperable transitional cell carcinoma of the urothelium (stage III and IV bladder cancer), (ii) autosomal dominant polycystic liver disease, (iii) autosomal dominant polycystic kidney disease, and (iv) noninfected painful total joint without mechanical complication (pseudotumor) [11]. A common feature of these pathologies is redox imbalance, accompanied by strong oxidative stress in the damaged tissue.

*Menadione and ascorbate are known to interfere with the mitochondrial electron transport chain* (ETC). Studies have demonstrated that menadione and other quinones affect mitochondrial respiration directly and even provide explanations of the molecular mechanisms for this mitochondrial interference [12–14]. For example, it has been demonstrated that menadione bypasses complex I deficiency [12, 14]. It is also known that pharmacological ascorbate and menadione are beneficial in the treatment of mitochondrial diseases [14, 15]. The combination of ascorbate and menadione is included in the *List of Dietary Supplements for Primary Mitochondrial Disorders* by the U.S. Department of Health and Human Services, National Institutes of Health (NIH). It has been used clinically to bypass complex III deficiency of the mitochondrial ETC [14, 15]. Menadione and ascorbate have been applied as a dietary supplement in combination with coenzyme Q10 (CoQ10), niacin, riboflavin, and thiamin to bypass complex I and complex III of the ETC [16]. Since menadione (in high concentrations) is hepatotoxic, it is no longer used in dietary supplements in the U.S., but still in use in other countries and common in animal feed (including diets for laboratory animals).

*Menadione and ascorbate are redox cyclers* and administered alone or in combination, they can induce intracellular production of ROS (mostly superoxide and hydrogen peroxide) by interaction with molecular oxygen [17, 18]. Ascorbate can be converted to semidehydroascorbate (semi-DHA) and dehydroascorbate (DHA) via one-electron and two-electron oxidation, respectively. These three forms are part of normal vitamin C metabolism. Menadione can be converted to semiquinone and menadiol by one-electron or two-electron reduction, respectively. Menadione is also known as provitamin K3 due to its conversion to vitamin K2 in mammals by bacteria and/or normal cell metabolism [19, 20]. Although M/A has often been referred to as a vitamin-based therapeutic strategy, this is a misnomer. It should be specified that M/A is not a vitamin. In fact, the redox-cycling anticancer effects of M/A do not appear to rely on the vitamin activities of these compounds. Rather, M/A is most correctly termed a combination drug.

It is generally accepted that the combination M/A causes cancer cell death by induction of oxidative stress and subsequent replicative stress [3, 4, 21–28]. However, the primary source of reactive oxygen species (ROS) and induction of severe oxidative stress in M/A-treated cancer cells is still unclear and is under discussion. The described potential mechanisms of the selective cytostatic/cytotoxic effects of M/A in cancer cells are not fully supported by the prior studies.

**Figure 1:**
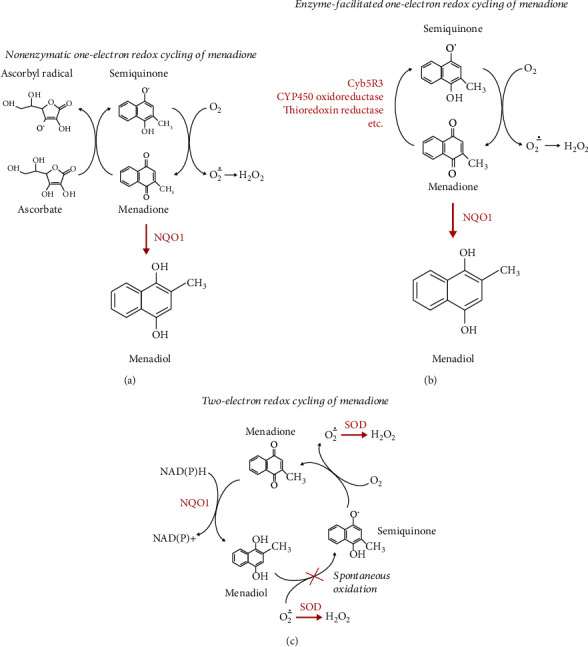
Schematic representation of redox cycling of menadione with production of superoxide and hydrogen peroxide: (a) nonenzymatic ascorbate-driven one-electron redox cycling; (b) enzyme-facilitated one-electron redox cycling; (c) two-electron redox cycling by NQO1 and subsequent autooxidation (according to R. Jabarak and J. Jabarak [30]). *Note*: NQO1 maintains menadione in its reduced form (menadiol) and thus depletes the menadione required for one-electron redox cycling mechanisms, while SOD converts superoxide into hydrogen peroxide, thus inhibiting spontaneous oxidation of menadiol to semiquinone. CYP450: cytochrome P-450; Cyb5R3: NADH-cytochrome b5 oxidoreductase 3; NQO1: NAD(P)H-dehydrogenase quinone 1; SOD: superoxide dismutase.

The most widely discussed mechanism postulated for M/A-mediated ROS production is nonenzymatic redox cycling between ascorbate and menadione (Figure 1(a)), which occurs in the extracellular space and cytosol [21, 29, 30]. This mechanism is proposed to explain the synergism between the two substances. However, in cells, overproduction of ROS is more likely to occur by enzymatically facilitated redox cycling of menadione. Enzyme-mediated reactions are faster and more favorable than nonenzymatic ascorbate-driven redox cycling (Figure 1(b)) [31]. Many flavin-containing and other enzymes (such as cytochrome P450 oxidoreductase, NADH-cytochrome b5 oxidoreductase 3 (Cyb5R3), and thioredoxin reductase) can reduce menadione to semiquinone [32–34]. Then, semiquinone is subsequently oxidized nonenzymatically with the production of superoxide [29, 30]. However, the enzymatic redox cycling of menadione does not explain the synergism between both substances. Moreover, in the cells, menadione should exist as menadiol, due to its NQO1-catalyzed two-electron reduction [30, 34–38]. Articles published so far in this field recognize the importance of NQO1 to M/A, but they do not discuss how menadione persists in the presence of this enzyme.

NQO1 is a two-electron reductase with a preference for short-chain quinones. It is considered to be a menadione detoxification enzyme (formerly termed menadione reductase or DT-diaphorase), which is upregulated (overexpressed) in various cancers [39, 40]. Therefore, the overproduction of ROS by enzymatic redox cycling of menadione is disputable if NQO1 is not inhibited (Figure 1(b)). This suggests the possibility of other mechanisms for the production of menadione and semiquinone apart from, or in addition to, the spontaneous oxidation of menadiol.

In cells, ascorbic acid exists mostly in reduced form due to the activity of two enzymes: (i) Cyb5R3, which converts semi-DHA to ascorbate by one-electron reduction, and (ii) glutathione peroxidase, which converts DHA to ascorbate by two-electron reduction [33, 41, 42].

We assume that the predominant existence of M/A in cells as menadiol/ascorbate excludes the interaction between both substances in real time, as well as the overproduction of ROS as a result of their nonenzymatic redox cycling (Figure 1(a)). Therefore, inside the cells, the one-electron redox cycling mechanisms of menadione are possible only if somehow menadiol can be oxidized by additional reactions.

R. Jabarak and J. Jabarak showed the ability of generating superoxide and semiquinone by the NQO1-catalized two-electron reduction of menadione to menadiol *in a cell-free system* (Figure 1(c)) [30]. However, their study demonstrates that the spontaneous nonenzymatic oxidation of menadiol to semiquinone is a very slow process. It requires superoxide and could be accelerated by ascorbate, but almost completely inhibited by superoxide dismutase (SOD). The authors have proposed two mechanisms for the superoxide production in this cell-free system: (i) a nonenzymatic redox cycling of ascorbate and menadione (reactions (1) and (2)) and (ii) a classical iron/ascorbate-dependent Fenton/Haber-Weiss mechanism (reactions (3) and (4)):

(1) $$\begin{array}{c} \text{ Q}+\text{Asc}{\text{H}}^{–}⟶{\text{Q}}^{\underline{•}}+\text{As}{\text{c}}^{\underline{•}\cdot }+{\text{H}}^{+} \end{array}$$

(2) $$\begin{array}{c} {\text{Q}}^{\underline{•}}+{\text{O}}_{2}⟶\text{Q}+{{{\text{O}}_{2}}^{\cdot }}^{\underline{•}}+{\text{H}}^{+} \end{array}$$

(3) $$\begin{array}{c} {\text{Fe}}^{3+}+\text{Asc}{\text{H}}^{–}⟶{\text{Fe}}^{2+}+\text{As}{\text{c}}^{\underline{•}}+{\text{H}}^{+} \end{array}$$

(4) $$\begin{array}{c} {\text{Fe}}^{2+}+{\text{O}}_{2}⟶{\text{Fe}}^{3+}+{{\text{O}}_{2}}^{\underline{•}} \end{array}$$

where Q is quinone (menadione), ${\text{Q}}^{\underline{•}}$ is semiquinone, AscH^−^ is ascorbate, and $\text{As}{\text{c}}^{\underline{•}}$ is semidehydroascorbate.

Thus, it can be assumed that in cell-free buffer systems, the two-electron redox cycling mechanism can produce a semiquinone for the one-electron redox cycling mechanisms. At the same time, the one-electron redox cycling mechanisms can yield a superoxide for the two-electron redox cycling mechanism. This is a reasonable explanation for the synergistic action of the two components. However, such interplay between mechanisms requires accumulation of superoxide sufficient to exceed the SOD capacity. NQO1 and SOD are commonly overexpressed in various types of cancers and generally associated with aggressive growth, although in many primary tumors Mn-SOD activity is markedly reduced [39, 40, 43, 44]. If this is the main mechanism for M/A-mediated cytotoxicity in cancer cells, it is inexplicable why there is no direct detection of overproduction of superoxide in M/A-treated cells. The data, published by other authors and suggesting that M/A destroys cancer cells by oxidative stress, are mainly indirect—based, for example, on attenuation of M/A-mediated cell death by precursors of glutathione, increase of end-products of free radical oxidation of biomacromolecules, and inhibition by exogenous catalase [21, 23, 25, 26, 28, 45].

*It should be noted that there is a gap between the in vivo and in vitro studies regarding the putative mechanism(s) of M/A-mediated anticancer effects*. In vivo, the therapeutic effects of M/A are thought to occur at relatively low plasma concentrations (≤2/200 *μ*M/*μ*M of M/A), attainable by oral administration, as well as at higher pharmacological concentrations, attainable by parenteral administration (intravenous (i.v.) or intraperitoneal (i.p.)) [1, 21, 22, 46–52]. Based on our knowledge, there are no in vitro studies on M/A-treated cultured cancer cells indicating cell death below 5/500 *μ*M/*μ*M of M/A. The already published data have demonstrated that M/A manifests significant cytotoxicity in high doses (≥10/1000 *μ*M/*μ*M) [49, 53, 54] via induction of apoptosis, ferroptosis, necrosis, and specific form of cell death termed “autoschizis” [55–65]. *It is curious why anticancer effects of M/A in vivo are demonstrated at significantly lower concentrations than those inducing cell death in vitro* [1, 3]. It was found that menadione and ascorbate, administered alone, manifest anti-inflammatory effects [66, 67] that also contribute to suppression of cancer progression and invasion [68, 69]. This suggests other therapeutic mechanisms of M/A beyond the direct oxidative killing of cancer cells, such as stimulation of immune recognition of malignant cells and sensitization to conventional therapy.

Our recent short communication shows that M/A induces overproduction of mitochondrial superoxide in cancer cells, but not in normal cells of the same origin [70]. Our further studies, described below, were directed to clarify the mechanism(s) of this phenomenon and its importance for the selective anticancer effect of M/A (details are given in *Supplementary Materials–*Appendix 1).

Experiments were designed (i) to clarify the role of mitochondria in the induction of oxidative stress in cancer cells treated with ascorbate, menadione, and their combination (M/A); (ii) to compare the cytotoxic effects of low/tolerable versus high doses, using both the reduced and oxidized forms of these small molecules; (iii) to compare the redox-modulating and cytotoxic potential of M/A on cancer and normal cells of the same origin; (iv) to further investigate the possibility for sensitizing cancer cells towards conventional anticancer drugs, using low/tolerable doses of M/A; and (v) to study the effect of M/A on the expression of programmed cell death ligand 1 (PD-L1) in cancer cells (one of the major immune checkpoints) and tumor growth in vivo.

*The emphasis is placed on the effects of low/tolerable concentration of M/A (1/100-3/300 μM/μM), attainable in cancer cells by oral and parenteral administration without significant side-effects on normal cells and tissues. In such doses, M/A could be an attractive adjuvant to cancer therapy.*

## 2. Materials and Methods

### 2.1. Chemicals

L-Ascorbate, (L)-dehydroascorbate, menadione, and menadiol were purchased from Sigma-Aldrich (Weinheim, Germany). Other chemicals and kits were purchased from Santa Cruz Biotechnology Inc., Sigma-Aldrich, Wako, Abcam, Promega, and Cell Biolabs Inc.

All reagents, used in the experiments, were “analytical grade” or “HPLC grade”.

### 2.2. Cells and Treatment Protocol

Most of the experiments were performed on leukemic lymphocytes (Jurkat; RIKEN Bioresource Center, Saitama, Japan), derived from patients with acute lymphoblastic leukemia, as well as on normal lymphocytes, derived from clinically healthy blood donors (Human Peripheral Blood Cells; Cell Applications Inc., USA).

This cellular model (leukemia/normal lymphocytes) has the following advantages: (i) relatively fast and easy isolation of normal lymphocytes (in large quantities) from peripheral blood of clinically healthy donors and (ii) rapid analysis of live/dead cells in suspensions after trypan blue staining and automatic counting (without loss of cells as in the case of adhesive cell lines); this model allows normalization of the data for each investigated biochemical parameter to the equal number of total/live cells (where appropriate), minimizing errors due to the loss of cells during washings and subsequent centrifugations before final measurements.

Normal lymphocytes were also isolated in our lab from peripheral blood of clinically healthy donors using Lymphosepar I (Immuno-Biological Laboratories Co., Fujioka, Japan) and multiple washing of the lymphocyte fraction by phosphate-buffered saline (PBS) solution. Multiple washing by PBS is obligatory to avoid contaminations of free and heme iron as a result of haemolysis during isolation. Any contaminations of transition metals in the cell fraction can compromise the results due to induction of Fenton's reactions in the presence of M/A.

Cells were cultured in RPMI-1640 medium (Sigma-Aldrich, Weinheim, Germany), supplemented with 10% heat-inactivated fetal bovine serum (Gibson, USA) and antibiotics (100 U/mL penicillin and 100 *μ*g/mL streptomycin) (Gibson, USA) in a humidified atmosphere at 37°C, saturated with 5% CO_2_. All cells were collected by centrifugation (1000 × *g*, 10 min, for leukemia lymphocytes and 1500 × *g*, 15 min, for normal lymphocytes) and placed in a fresh medium without antibiotics prior to treatment with the respective substance. The cells (1 × 10^6^ cells/mL) were incubated with ascorbate, menadione, and their combination for different time intervals in a cell incubator. At each time interval, aliquots were used for analyses.

Experiments were also performed on adhesive cells lines: (i) colon epithelial cells—cancer (Colon26) and normal (FHC) (Cell Applications Inc., San Diego, USA) and (ii) breast epithelial cells—cancer (MFC7) and normal (MCF10A) (Cell Applications Inc., San Diego, USA). Colon26 and MCF7 were cultured in DMEM (Sigma-Aldrich, Weinheim, Germany). FHC and MCF10A were cultured in DMEM-F12 (Sigma-Aldrich, Weinheim, Germany) and DMEM, respectively, both supplemented with growth factors. All mediums were supplemented with 10% FBS (heat inactivated) (Gibson, USA). Twenty four hours before the experiment, the cells were replaced in a fresh medium without growth factors. To remove the adhesive cells from the plates, we used a trypsin-EDTA solution (0.5% of trypsin, 0.2% of EDTA) and subsequent washing with PBS. During the culturing and experiments, the adhesive cells were sedimented by centrifugation (800 × g/5 min). The concentration of glucose in the cell cultured mediums was standard (2 mM).

Ascorbate was dissolved in phosphate-buffered saline (PBS; 10 mM, pH 7.4). Menadione, menadiol, and dehydroascorbate were dissolved in DMSO to 50 mM stock solution, and then, several working solutions in PBS were prepared. The final concentration of DMSO in the cell suspension was below 1%. At this concentration, DMSO did not influence cell viability. The cells were washed with 10 mM PBS (pH 7.4), if necessary, depending on biochemical analysis.

### 2.3. Cell Proliferation and Viability Assays

Cell viability and proliferation were analyzed by trypan blue staining and automated counting, using a Countess™ Automated Cell Counter (Invitrogen, Oregon, USA).

Briefly, 10 *μ*L of trypan blue (0.4%) was added to 10 *μ*L of cell suspension, incubated for 30 s, and 10 *μ*L of the cell suspension was placed in a Countess® (Invitrogen) glass chamber. The number of live and dead cells in the suspension was counted automatically. The linear range to operate with the automated cell counter was 1 × 10^4^–5 × 10^6^ cells/mL, and the optimal cell size was in the range of 5-60 *μ*m.

### 2.4. Dihydroethidium (DHE) Assay

DHE is a cell-penetrating fluorogenic probe, interacting predominantly with superoxide [71]. It allows distinguishing between superoxide and hydrogen peroxide and analyzing the level of intracellular superoxide.

Briefly, DHE was dissolved in DMSO to 65 mM stock solution (kept at -40°C, for one month, aliquoted), which was diluted with PBS to prepare 300 *μ*M of DHE working solution in the day of experiment. 10 *μ*L of DHE (300 *μ*M) was added to 1 mL of cell suspension (1 × 10^6^ cells/mL; the final DHE concentration was 3 *μ*M). The samples were incubated for 30 min min at room temperature, protected from light, washed three times with PBS using centrifugation, and finally resuspended in 1 mL of PBS. The fluorescence intensity was detected immediately at *λ*_ex_ = 518 nm and *λ*_em_ = 605 nm, using a microplate reader (TECAN Infinite® M1000, Austria).

### 2.5. MitoSOX Assay

MitoSOX™ Red Mitochondrial Superoxide Indicator (Molecular Probes, Invitrogen, USA) is a fluorogenic probe for highly selective detection of superoxide in the mitochondria of live cells. The probe is a DHE derivate, containing triphenylphosphonium group. Once in the mitochondria, the MitoSOX™ Red reagent is oxidized by superoxide and exhibits red fluorescence [71, 72]. The probe is not oxidized by other ROS/RNS, and its oxidation is prevented by superoxide dismutase [72].

Briefly, MitoSOX™ Red was dissolved in DMSO to 5 mM stock solution, which was diluted with Hank's Balanced Salt Solution (HBSS, containing Ca^2+^ and Mg^2+^) to prepare 3 *μ*M MitoSOX™ Red working solution on the day of experiment. 1 mL of cells (1 × 10^6^ cells/mL for nonadhesive cells and 5 × 10^5^ cells/mL for adhesive cells) was collected by centrifugation, and the pellet was resuspended in 1 mL of 3 *μ*M MitoSOX™ Red. The samples were incubated for 30 min at room temperature, protected from light, washed three times with PBS using centrifugation, and finally resuspended in 1 mL of PBS. The fluorescence intensity was detected immediately at *λ*_ex_ = 510 nm and *λ*_em_ = 580 nm, using a microplate reader (TECAN Infinite® M1000, Austria) or fluorescence confocal microscope (live cell imaging; magnification 60x) (Olympus DP73, Japan).

### 2.6. Dihydrodichlorofluorescein (DCF) Assay

2,7-Dichlorodihydrofluorescein diacetate (DCFH-DA) is a cell-penetrating fluorogenic probe, interacting predominantly with hydrogen peroxide [71]. It allows analyzing the level of intracellular hydroperoxides, using OxiSelect™ ROS Assay Kit (Green Fluorescence) (Cell Biolabs, Inc., US).

Briefly, 500 *μ*L of cells (1 × 10^6^ cells/mL) were placed in 12-well plates. 50 *μ*L of 1 mM DCFH-DA was added to each well. The samples were incubated for 60 min at room temperature, washed three times with PBS using centrifugation, and finally resuspended in 500 *μ*L of PBS. The fluorescence intensity was detected immediately at *λ*_ex_ = 480 nm and *λ*_em_ = 530 nm, using a microplate reader (TECAN Infinite® M1000, Austria). DCF was used as a standard.

### 2.7. ATP Assay

Intracellular ATP was analyzed using the CellTiter-Glo™ luminescent cell viability assay (Promega). The analysis is based on generation of a luminescent signal from luciferin/luciferase reaction, which is proportional to the amount of ATP synthesized in live cells [73].

Briefly, 90 *μ*L aliquots of cell suspensions (1 × 10^6^ cells/mL) were placed in 96-well plates and incubated with M/A for 24 and 48 hours, in humidified atmosphere (at 37°C, 5% CO_2_). 100 *μ*L of CellTiter-Glo reagent (containing luciferin and luciferase) was added to each well, followed by incubation using the protocol recommended by the manufacturer. The luminescence, produced by the luciferase-catalyzed conversion of luciferin into oxyluciferin in the presence of ATP, was detected using a microplate reader (TECAN Infinite® M1000, Austria), working in a chemiluminescent mode.

### 2.8. Mitochondrial Membrane Potential

Mitochondrial membrane potential was analyzed using tetramethylrhodamine methyl ester (TMRE) as described in Levraut et al. [74] with slight modifications. TMRE is a cell-penetrating, cationic fluorophore, which accumulates in the mitochondrial matrix based on mitochondrial membrane potential. The fluorescence intensity is proportional to the mitochondrial potential and decreases upon depolarization of the mitochondrial membrane.

Briefly, 1000 *μ*L of cells (1 × 10^6^ cells/mL) was placed in 12-well plates. 5 *μ*L of TMRE (from 40 *μ*M stock solution in DMSO) was added to each well. The samples were incubated at 37°C for 30 min, washed twice with PBS using centrifugation, and finally resuspended in 500 *μ*L of PBS. The fluorescence intensity was detected immediately at *λ*_ex_ = 550 nm and *λ*_em_ = 575 nm, using a microplate reader (TECAN Infinite® M1000, Austria).

### 2.9. Succinate Assay

The succinate level was analyzed using the Succinate Assay Kit (Colorimetric) (Abcam, Japan). The analysis is based on a coupled enzyme reaction, which results in a colored product with absorbance maximum at 450 nm, proportional to the succinate concentration in the sample. Succinate was used as a standard.

Briefly, cells (1 × 10^6^ cells per sample) were lysed in 100 *μ*L of succinate assay buffer as described in the manufacturer's instructions. 50 *μ*L (in duplicates) of each cell lysate was placed in a 96-well plate and incubated with 50 *μ*L of reaction mix 1 or 50 *μ*L of reaction mix 2 (without succinate converter; blank sample) for 20 min at 37°C, in the dark. Absorbance at 450 nm was recorded, using a microplate reader (TECAN Infinite® M1000, Austria). Blank sample was included to correct the NADH-dependent background absorbance.

### 2.10. NAD^+^/NADH Quantification Assay

The NAD^+^/NADH level was analyzed using the NAD^+^/NADH Quantification Kit (Sigma-Aldrich, St. Louis, MO, USA). The assay is specific for NAD^+^ and NADH and does not detect NADP^+^ and NADPH. NAD_total_ and NADH are quantified spectrophotometrically at 450 nm.

Briefly, cells (2 × 10^5^ cells per sample) were placed in 100 *μ*L NAD^+^/NADH extraction buffer and homogenized as described in the manufacturer's instructions. Cell lysates were purified on 10 kDa cut-off spin filter. 50 *μ*L (in duplicates) of each sample was placed in a 96-well plate and incubated with 100 *μ*L of master reaction mix for 5 min at room temperature to convert NAD^+^ to NADH (for NAD_total_ determination). 10 *μ*L of NADH developer was added to each sample and incubated for 1 h at room temperature. Absorbance at 450 nm was recorded, using a microplate reader (TECAN Infinite® M1000, Austria). For detection of NADH only, aliquots of cell lysates were placed in a heating block for 30 min at 60°C, to decompose NAD^+^, before proceeding to analysis.

### 2.11. Apoptosis Assay

The induction of apoptosis was analyzed by the expression of phosphatidylserine (PSer) on the cell surface, using the FITC-Annexin V Apoptosis Detection Kit (BioVision, Milpitas, CA, USA).

Briefly, the cells (1 × 10^6^ cells/mL) were incubated with conventional anticancer drug, vitamin C plus K3, or their combination. At different time points, the cells were collected by centrifugation (1000 × *g*, 10 min), washed twice with PBS containing 2.5 mM CaCl_2_ (Annexin V-binding buffer), and resuspended in the same buffer. One hundred microliters of the suspension was incubated with 5 *μ*L of fluorescein isothiocyanate (FITC)-Annexin V for 10 min at room temperature in the dark. The cells were washed three times with Annexin V-binding buffer and were finally resuspended in 500 *μ*L of the same buffer. FITC-Annexin V bound to PSer exposed on the cell surface was detected spectrofluorimetrically at *λ*_ex_ = 488 nm and *λ*_em_ = 535 nm, using a Tecan Infinite F200 PRO (Tecan Austria GmbH) microplate reader.

### 2.12. Total Glutathione Assay

The total glutathione (GSH/GSSG) in cell suspensions (5 × 10^6^ cells/mL) was analyzed by the OxiSelect™ Total Glutathione (GSSG/GSH) Assay Kit (Cell Biolabs, Inc., US) as it was described in the manufacturer's instruction. The method is based on reduction of GSSG to GSH by glutathione reductase in the presence of NADPH and subsequent addition of chromogen. Chromogen reacts with the thiol group of GSH with production of spectrophotometrically detectable compound at 405 nm, using a Tecan Infinite F200 PRO (Tecan Austria GmbH) microplate reader. The total glutathione content in the cell suspension was determined by a calibration curve using a glutathione standard.

### 2.13. EPR Spectroscopy

EPR experiments were performed on an X-Band spectrometer (JOEL, Tokyo, Japan) with standard cavity, at the following parameters: microwave frequency: 9.4 GHz, field strength = 336 mT, field modulation frequency = 100 kHz, field modulation amplitude = 0.063 mT, microwave power = 2.0 mW, time constant = 0.01 s, sweep width = 10 mT, scan time (sweep time) = 1 min, and number of scans = 1. In all EPR experiments, cells were collected by centrifugation and resuspended in medium without antibiotics.

### 2.14. FACS Analysis of PD-L1 Expression in Cells

FACS analysis of PD-L1 expression in cultured B16 melanoma cells was performed by Crown Bio (Taicang) Inc. (Beijing, China), using BD C6 FACS (BD, USA) equipment. Cells were harvested during the logarithmic growth period and counted. Cell concentrations were adjusted to the appropriate number with medium, and 5 mL of cell suspensions was added to T25 flasks. Cells were incubated overnight in humidified atmosphere (at 37°C, 5% CO_2_) and after that treated with M/A in different concentrations. Nontreated cells were used as controls. The incubation in the absence and presence of M/A was carried out for 72 hours in a cell incubator. Accutase™ cell detachment solution (Sigma-Aldrich) and low-speed centrifugation (300 × g, 5 min) were used to collect the cells after incubation. Cells were washed twice with fresh medium and replaced into labeled FACS tubes for PD-L1 staining, using anti-PD-L1 antibody. In parallel, 7-aminoactinomycin D (7-AAD) staining was used for live cell imaging. Rat IgG2b-kappa was used as an isotype control.

### 2.15. Melanoma Model in Mice

Female C57BL/6N mice were obtained from Charles River Labs (Japan). All mice were female and were used at 6 weeks of age and maintained in specific pathogen-free conditions. The animal experiments in this study were approved by the National Institute of Radiological Sciences Institutional Animal Care and Use Committee, and all experiments were performed in accordance with relevant guidelines and regulations (Protocol No. 19-1201).

B16F10-OVA (B16F10 melanoma transduced to express OVA) were cultured in RPMI-1640 supplemented with 10% FCS, 2 mmol/L L-glutamine, 0.05 mmol/L 2-mercaptoethanol, HEPES, penicillin, and streptomycin at 37°C in a humidified atmosphere containing 5% CO_2_.

B16F10-OVA cells (5 × 10^5^ per mouse) were intradermally (i.d.) inoculated into the right hind limb. The tumor-bearing mice in each cage were randomly assigned to the following three groups: (i) untreated group (control group, *n* = 5), (ii) menadione/ascorbate- (M/A) treated group, i.p. injection of 0.5 g/kg and 5 mg/kg, respectively (IP group, *n* = 5), and (iii) M/A-treated group, oral administration of 15 g/L and 150 mg/L, respectively (OA group, *n* = 5). From two days before injecting M/A into the tumor-bearing mice, the mice were fed the vitamin C- and K3-deficient diet (CLEA, Tokyo, Japan). When the tumor size became about 3–5 mm in diameter at 7 days following tumor inoculation, both intraperitoneal and oral administration of M/A began. The mice in the IP group were administered a fresh M/A solution every day except on weekends, and the mice in the OA group were allowed to freely drink a fresh M/A solution every day. Body weights were measured every week. The tumor volume was calculated by the following formula: tumor volume (mm^3^) = 0.5 × length (mm) × [width (mm)]^2^.

### 2.16. Statistical Analysis

All results are expressed as means ± standard deviation (SD). The normality of the distribution for all parameters of each experimental sample in vitro, as well as each experimental group in vivo, was initially confirmed by using the Kolmogorov-Smirnov test. The most extreme differences for all experimental samples/groups were below the critical *D* values. Based on the normality of distribution in all samples/groups, the comparisons between them were performed using Student's *t*-test and Bonferroni's test for multiple comparisons. Two-tailed *p* values of less than 0.05 were considered statistically significant.

## 3. Results

### 3.1. Dose-Dependent Effects of Menadione/Ascorbate on Cell Proliferation and Viability: Cytostatic versus Cytotoxic Effect

**Figure 2:**
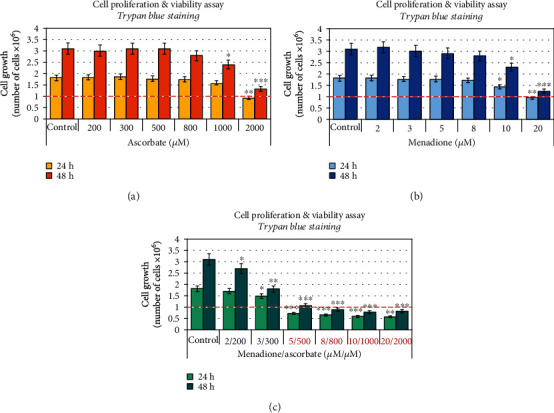
Effects of ascorbate, menadione, and their combination on proliferation of leukemic lymphocytes, after 24 and 48 hours of incubation in humidified atmosphere, analyzed by trypan blue staining and automated cell counting. The number of cells in all samples at the beginning of each experiment was 1 × 10^6^ cells/mL. Before treatment, cell viability was 98 ± 2%, analyzed by trypan blue staining. After treatment, cell viability was the same, except in the case of ascorbate-treated (2000 *μ*M), menadione-treated (20 *μ*M), and menadione/ascorbate-treated (5/500, 8/800, 10/1000, and 20/2000 *μ*M/*μ*M) cell suspensions, where the cell viability was 85 ± 5%, analyzed by trypan blue staining. The data are means ± SD from eight independent experiments for (a) and (b) and twelve independent experiments for (c). ∗*p* < 0.05, ∗∗*p* < 0.01, and ∗∗∗*p* < 0.001 versus the respective control (untreated cells).

Ascorbate, menadione, and their combination decreased the proliferation activity of leukemic lymphocytes in a concentration-dependent and time-dependent manner (Figure 2). The data were obtained using trypan blue staining and automated counting of live/dead cells. Ascorbate (≤800 *μ*M) and menadione (≤8 *μ*M), administered separately, did not affect cell proliferation and viability (Figures 2(a) and 2(b)). However, the combination M/A exhibited a synergistic antiproliferative effect at concentrations ≥ 2/200 *μ*M/*μ*M, especially at 48-hour incubation (Figure 2(c)). M/A decreased cell viability at concentrations ≥ 5/500 *μ*M/*μ*M. In this case, the number of cells in all treated samples for 24 hours was below the initial number at the beginning of the experiment (1 × 10^6^ cells/mL), which indicates a cytotoxic effect in addition to a cytostatic effect.

**Figure 3:**
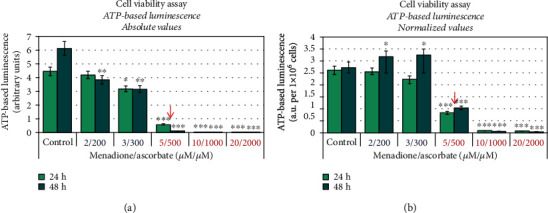
Effects of menadione/ascorbate on proliferation activity of leukemic lymphocytes, after 24 and 48 hours of incubation in humidified atmosphere, analyzed by ATP-based luminescence (CellTiter-Glo™ Luminescent Cell Viability Assay). The proliferation activity of the treated cells was presented as ATP-based luminescence in (a) absolute values (arbitrary unites) and (b) normalized to equal number of living cells in each sample (1 × 10^6^ cells/mL). The data are means ± SD from three independent experiments. The red arrow indicates the concentration to which the effect of menadione/ascorbate on ATP-based luminescence was reversible. ∗*p* < 0.05, ∗∗*p* < 0.01, and ∗∗∗*p* < 0.001 versus the respective control (untreated cells).

The viability of M/A-treated cells was also analyzed by CellTiter-Glo® Luminescence Cell Viability Assay, which is based on the ATP synthesis by the living cells (Figure 3). The results are presented as (i) ATP-based luminescence in absolute units (Figure 3(a)) and (ii) ATP-based luminescence, normalized to equal number of living cells in each sample (Figure 3(b)), since mainly living cells produce ATP. ATP-based luminescence is characterized by interesting dynamics. The trend was the same as for the trypan blue test when the data are presented in absolute units—ATP-based luminescence decreased in M/A-treated cells, in a concentration-dependent manner (Figure 3(a)). By normalizing the data to an equal number of living cells, two opposite trends were observed (Figure 3(b)):

- At high concentrations of M/A (≥10/1000 *μ*M/*μ*M), luminescence decreases with time of incubation. This indicates a decrease in the number of living cells in the suspensions, suggesting a cytotoxicity of M/A
- At low/tolerable concentrations of M/A (≤5/500 *μ*M/*μ*M), the luminescence decreases in a concentration-dependent manner after 24 hours of incubation. However, at 48 hours of incubation with 2/200 and 3/300 *μ*M/*μ*M of M/A, the luminescence is above the control level. In the case of 5/500 *μ*M/*μ*M of M/A-treated cells, it is below the control level, but slightly above the level detected at 24 hours of incubation

The data in Figures 2(c) and 3 could be interpreted in two aspects:

- Low/tolerable concentrations of M/A do not significantly affect the steady-state levels of ATP in Jurkat cells, but the same concentrations decrease proliferative activity, based on both assays
- A concentration of 5/500 *μ*M/*μ*M of M/A is the critical concentration for the transition from cytostatic to a cytotoxic effect in the same cells

It should be noted that ascorbate and menadione, applied separately in the respective concentrations, did not affect the ATP-based luminescence obtained from the luciferin/luciferase reaction in a model (cell-free) system. Therefore, both substances do not cause artifacts in the analysis, as it is in the case of methyl tetrazolium- (MTT/MTS) based methods.

It is well known that redox-active substances (such as ascorbic acid, vitamin A, and sulfhydryl-containing compounds) induce a direct nonenzymatic reduction of the MTT/MTS to formazan [75, 76]. Chemicals that uncouple electron transport from oxidative phosphorylation of ATP also are known to interfere with the MTT/MTS assay [77]. In addition, the MTT/MTS assay does not allow evaluation of the real cytotoxic effects of ascorbate, menadione, and their combination. This is due to the impossibility of quantifying living/dead cells in the suspensions, compared to the initial number of cells at the beginning of treatment.

The choice of analytical test and data processing are crucial for correct interpretations and conclusions. The use of more than one analytical test based on different approaches, such as microscopic and biochemical, as well as the choice of appropriate data processing, contributes to the elimination and minimization of artifacts and misinterpretations. Thus, comparative analysis of the data in Figures 2 and 3 shows that low/tolerable concentrations of M/A (≤3/300 *μ*M/*μ*M) slightly suppress the proliferation of leukemic lymphocytes (in particular, Jurkat), but do not decrease the steady-state levels of ATP. A M/A concentration of 5/500 *μ*M/*μ*M is an inflection concentration for recovery of ATP levels in the cells. This suggests that ATP depletion in M/A-treated cells could be a result of suppression of mitochondrial respiration. A menadione concentration of 5 *μ*M is considered crucial for its mitochondrial redox cycling [14, 74, 78]. Chan et al. have reported that complex I bypass and ATP recovery in menadione-treated cells occurs only at concentrations below 5 *μ*M and it is considered a threshold level [14]. This suggests that ATP depletion in M/A-treated cells could be a result of suppression of mitochondrial respiration at concentrations over 5/500 *μ*M/*μ*M.

### 3.2. Dose-Dependent Effects of Menadione/Ascorbate on Mitochondrial Superoxide in Cancer and Normal Cells of the Same Origin: Correlation with Cell Viability

It is generally accepted that M/A suppresses proliferation and triggers cell death through overproduction of ROS, severe oxidative stress, and irreversible redox imbalance [1, 3, 21–28]. Two sources of M/A-induced oxidative stress have been discussed in the literature: (i) extracellular and (ii) intracellular—cytosolic. Both mechanisms are based on the assumption of overproduction of hydrogen peroxide due to one-electron menadione/ascorbate redox cycling [23–25, 28, 30, 57, 79]. All these events are reported at high concentrations of M/A (>5/500 *μ*M/*μ*M), and conclusions are based on indirect evidence such as (i) effects of catalase, metal chelators, antioxidants, and end-products of oxidative stress in M/A-treated cells [23–25, 28, 57, 65] or (iii) production of superoxide and/or hydrogen peroxide in cells treated with menadione or ascorbate alone [79–84]. Based on our knowledge, there are no data about direct detection of production and/or degradation of superoxide and/or hydrogen peroxide in M/A-treated cells.

Our efforts are aimed at clarifying the role of mitochondria in triggering oxidative stress in M/A-treated cancer cells, as well as the role of each substance individually at different concentrations (within the ranges of 2-20 *μ*M for menadione and 200-2000 *μ*M for ascorbate). For this purpose, we used two sensors for mitochondrial ROS and in particular for mitochondrial superoxide: (i) fluorogenic probe MitoSOX and (ii) cyclic hydroxylamine mito-TEMPOH. Both probes penetrate the cells and localize mainly in the mitochondria [71, 72]. Signals from the probes are not detected in the absence of superoxide, but are detectable after their interaction with superoxide, which was visualized by the appearance of fluorescence signal (in the presence of MitoSOX) or EPR signal (in the presence of mito-TEMPOH) in the cell suspensions [71, 72, 85, 86].

**Figure 4:**
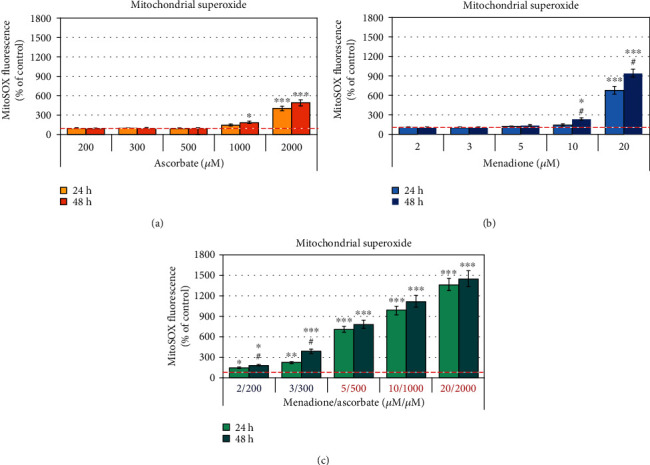
Effects of (a) ascorbate, (b) menadione, and (c) their combination on the level of mitochondrial superoxide in leukemic lymphocytes, after 24- and 48-hour incubation in humidified atmosphere, analyzed by MitoSOX fluorescence. The experimental conditions are the same as in Figure 2. MitoSOX fluorescence of treated cells was presented as a percentage of the MitoSOX fluorescence of control (untreated cells), which was considered 100%. The red dotted lines indicate the level of MitoSOX fluorescence in the control. The data are means ± SD from three independent experiments for (a) and (b) and eight independent experiments for (c). ∗*p* < 0.05, ∗∗*p* < 0.01, and ∗∗∗*p* < 0.001 versus the respective control (untreated cells). ^#^Statistically significant versus the respective M/A-treated sample for 24 hours.

High concentrations of ascorbate significantly increased mitochondrial superoxide (~1.5-2 times at 1000 *μ*M and ~4-5 times at 2000 *μ*M of ascorbate) (Figure 4(a)). At concentrations ≤ 500 *μ*M, ascorbate did not affect the level of mitochondrial superoxide. Similar data were obtained with menadione (Figure 4(b)). Mitochondrial superoxide increased in cells treated with high concentrations of menadione (~1.5-2 times at 10 *μ*M and ~7-9 times at 20 *μ*M of menadione). The level of mitochondrial superoxide increased significantly (~2 times) at 10 *μ*M of menadione for 48-hour incubation, but it was at the baseline at lower concentrations of menadione. *Surprisingly, an impressive, dose-dependent increase of mitochondrial superoxide was observed in M/A-treated leukemic lymphocytes (*Figure 4(c)*). The level of mitochondrial superoxide increased from 1.5-2 times at 2/200 μM/μM of M/A to ~15 times at 20/2000 μM/μM of M/A.*

Similar data were obtained by the EPR analysis of mitochondrial superoxide using mito-TEMPOH as a molecular sensor (Figure 1S in Supplementary Materials).

**Figure 5:**
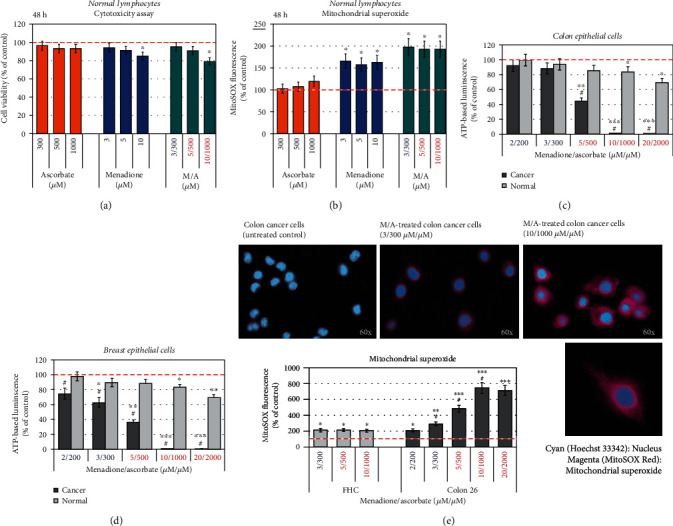
(a) Effects of ascorbate, menadione, and their combination menadione/ascorbate (M/A) on viability of normal lymphocytes, analyzed after 48 hours of incubation using trypan blue staining and automated cell counting. (b) Effects of ascorbate, menadione, and M/A on the level of mitochondrial superoxide in normal lymphocytes, analyzed after 48 hours of incubation using MitoSOX fluorescence. (c, d) Effects of M/A on the viability of cancer and normal epithelial cells, after 24 hours of incubation in humidified atmosphere, analyzed by ATP-based luminescence: (c) cancer and normal colon epithelial cells (Colon26 and FHC, respectively); (d) cancer and normal mammary epithelial cells (MCF7 and MCF10A, respectively). (e) Effects of M/A on the level of mitochondrial superoxide in normal (FHC) and cancer (Colon26) colon epithelial cells, analyzed after 24 hours of incubation using MitoSOX™ Red Mitochondrial Superoxide Indicator and fluorescence spectroscopy and microscopy. The number of cells in all samples at the beginning of each experiment was 1 × 10^6^ cells/mL for (a) and (b) and 5 × 10^5^ cells/mL for (c)–(e). All data are presented as a percentage of the respective control (untreated cells), which was considered 100%. The red dotted lines indicate the level of control. The data are means ± SD from three independent experiments. Representative fluorescence images are shown in (e). In all charts, ∗*p* < 0.05, ∗∗*p* < 0.01, and ∗∗∗*p* < 0.001 versus the respective control (untreated cells). In the charts of (c)–(e): ^#^statistically significant versus the respective M/A-treated normal cells.

We next sought to clarify whether the effects of ascorbate, menadione, and their combination are specific to cancer cells, or do these substances also affect the normal cells. Normal lymphocytes were incubated for 48 hours with menadione and/or ascorbate and two parameters were analyzed: (i) cell viability (Figure 5(a)) and (ii) mitochondrial superoxide (Figure 5(b)). Ascorbate did not affect cell viability and mitochondrial superoxide up to 1000 *μ*M. Menadione exhibited a slight cytotoxicity at 10 *μ*M (~15%) and a slight increase in mitochondrial superoxide (~50%) in a concentration-independent manner. In normal cells, the M/A combination exhibited similar effects as menadione—no effect on cell viability up to 5/500 *μ*M/*μ*M, a slight cytotoxicity (~20%) at 10/1000 *μ*M/*μ*M, and about 2 times increase of mitochondrial superoxide in a concentration-independent manner, which seems to be well tolerated.

Similar data were obtained on normal and cancer colon epithelial cells, as well as on normal and cancer breast epithelial cells, treated with M/A in different concentrations (Figures 5(c) and 5(d)). In concentrations ≥ 5/500 *μ*M/*μ*M, M/A exhibited a strong cytotoxicity towards cancer epithelial cells, but not towards normal epithelial cells. This is accompanied by a strong increase of mitochondrial superoxide in M/A-treated cancer cells (Figure 5(e)). The images from fluorescence microscopy, using MitoSOX as mitochondrial superoxide sensor, are very indicative.

The comparative analysis of the data obtained on cancer and normal cells shows that M/A exhibits selective cytotoxicity towards cancer cells without significant effect on the viability of normal cells of the same origin. This is accompanied by a very strong production of mitochondrial superoxide in M/A-treated cancer cells in a concentration-dependent manner (up to ~8-15 times above the baseline level), but not in normal cells. This could be the basis of the selective anticancer effect of M/A.

The higher susceptibility of cancer cells to M/A could be also explained by the higher ascorbate and/or menadione uptake compared to normal cells [67, 87, 88]. It is well known that vitamin C transporters are overexpressed on the plasmatic and outer mitochondrial membranes of many types of cancer cells [67, 87, 88].

### 3.3. Effects of Oxidized and Reduced Forms of Vitamins C and K3 on Cell Viability: Comparative Analysis

**Figure 6:**
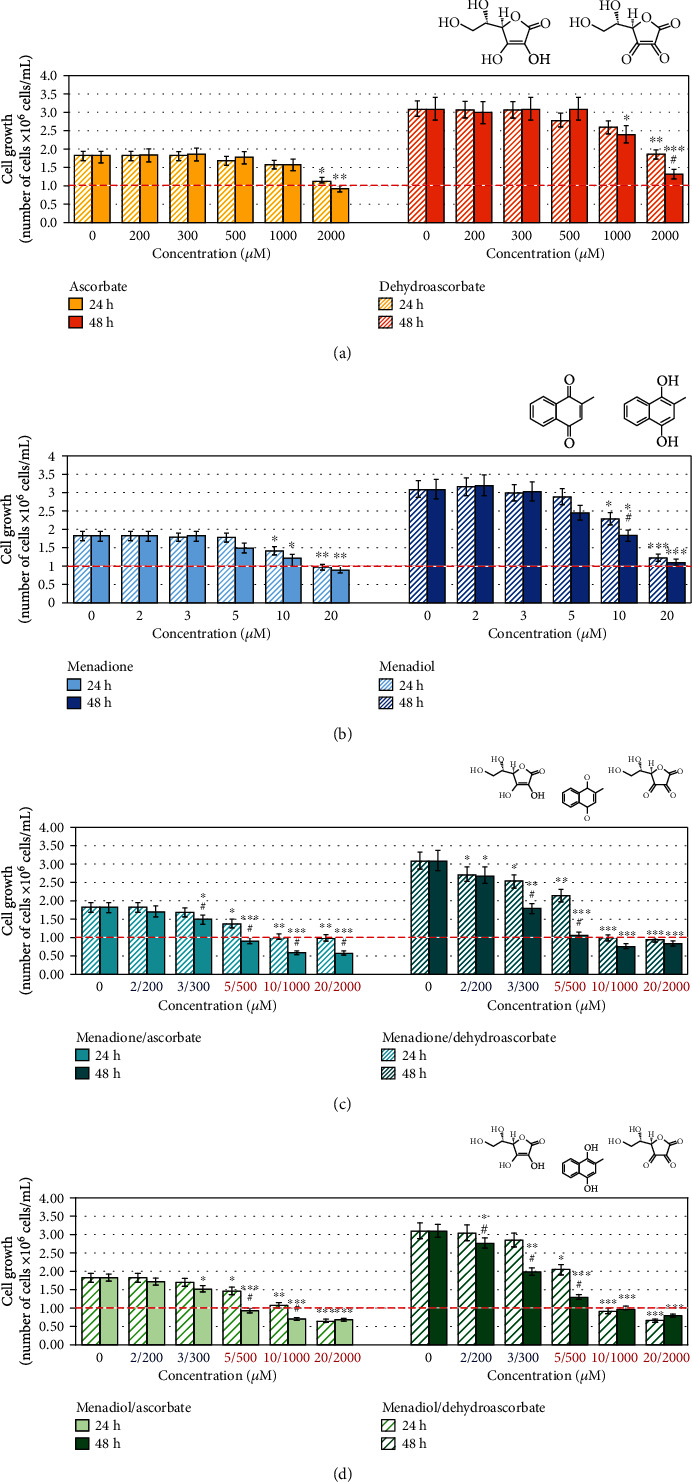
Effect of menadione, menadiol, and their combinations with ascorbate and dehydroascorbate on proliferation activity of leukemic lymphocytes, after 24 and 48 hours of incubation in humidified atmosphere, analyzed by trypan blue staining and automated cell counting: (a) ascorbate versus dehydroascorbate; (b) menadione versus menadiol; (c) menadione/ascorbate versus menadione/dehydroascorbate; (d) menadiol/ascorbate versus menadiol/dehydroascorbate. The proliferation activity of the treated cells was presented as a percentage of the proliferation activity of control (untreated cells), which was considered 100%. The number of cells in all samples at the beginning of each experiment was 1 × 10^6^ cells/mL. The red dotted lines indicate the initial number of cells. The data are means ± SD from four independent experiments for all charts. ∗*p* < 0.05, ∗∗*p* < 0.01, and ∗∗∗*p* < 0.001 versus the respective control (untreated cells). In (a): ^#^*p* < 0.05 versus the sample, treated with an equal amount of DHA. In (b): ^#^*p* < 0.05 versus the sample, treated with an equal amount of menadiol. In (c): ^#^*p* < 0.05 versus the sample, treated with an equal amount of menadione/DHA. In (d): ^#^*p* < 0.05 versus the sample, treated with an equal amount of menadiol/DHA.

**Figure 7:**
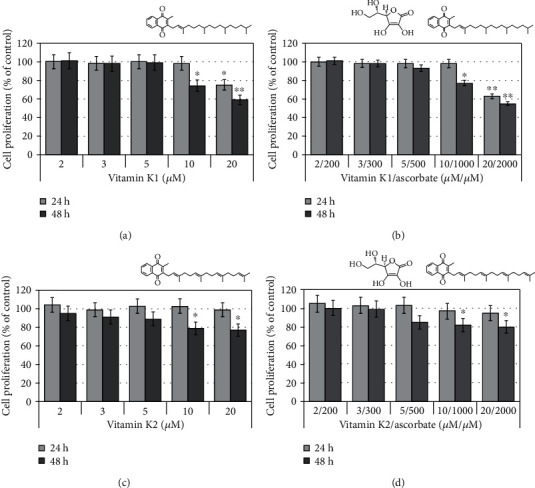
Effect of vitamin K2 (menaquinone-4), vitamin K1 (phylloquinone), and their combinations with ascorbate on proliferation activity of leukemic lymphocytes, after 24 and 48 hours of incubation in humidified atmosphere, analyzed by trypan blue staining and automated cell counting: (a) vitamin K1; (b) vitamin K1/ascorbate; (c) vitamin K2; (d) vitamin K2/ascorbate. The proliferation activity of the treated cells was presented as a percentage of the proliferation activity of control (untreated cells), which was considered 100%. The number of cells in all samples at the beginning of each experiment was 1 × 10^6^ cells/mL. The data are means ± SD from three independent experiments. Before the treatment, cell viability was 98 ± 2%, and after the treatment, it was 95 ± 3%. ∗*p* < 0.05, ∗∗*p* < 0.01, and ∗∗∗*p* < 0.001 versus the respective control (untreated cells).

To clarify whether or not the synergistic antiproliferative effect is unique to the combination menadione/ascorbate, we analyzed the combinations of other oxidized and reduced forms of vitamin C and provitamin K3 (Figure 6), as well as the combinations of ascorbate with vitamins K2 and K1 (Figure 7).

Dehydroascorbate (<2000 *μ*M) showed a similar antiproliferative effect on leukemic lymphocytes as ascorbate (Figure 6(a)). The antiproliferative effect of menadiol (in concentrations ≥ 5 *μ*M) was slightly but insignificantly lower than that of menadione (Figure 6(b)). There was no significant difference in the effects of menadione/ascorbate and menadiol/ascorbate on cell proliferation and viability (Figures 6(c) and 6(d), solid columns). In both combinations, very well expressed synergistic antiproliferative effects were detected within 2/200-10/1000 *μ*M/*μ*M, especially at 48 hours of incubation. The effects of menadione/dehydroascorbate and menadiol/dehydroascorbate on cell proliferation were also similar (Figures 6(c) and 6(d), striped columns).

The difference between combinations with ascorbate and dehydroascorbate can be explained by (i) higher instability of dehydroascorbate and its degradation to 2,3-diketogulonate in the cultured medium (this process also occurs in the bloodstream) [89, 90], (ii) extracellular mechanism for ascorbate-mediated generation of hydrogen peroxide [67, 90, 91], which does not exist for dehydroascorbate, and (iii) its interaction with the mitochondrial mechanism(s) for M/A-induced overproduction of superoxide, described in our study (Figures 4 and 5(e)).

Menadione/ascorbate and menadiol/ascorbate manifested similar antiproliferative and cytotoxic effects (Figures 5(c) and 5(d), solid columns). This suggests that the hydrogen peroxide, which is generated as a result of the extracellular redox cycling between menadione and ascorbate, is not a decisive factor for the antiproliferative effect of their combination, especially at low/tolerable concentrations. We found that preincubation of M/A-treated cells with catalase for 1 hour or 6 hours slightly diminished the antiproliferative and cytotoxic effect of high concentrations of M/A for 24 hours of incubation, but not for 48 hours of incubation (Figure 2S in *Supplementary Materials*).

The combinations vitamin K1/ascorbate and vitamin K2/ascorbate did not show cytotoxic effects on leukemic lymphocytes (Figures 7(b) and 7(d)). In concentrations ≥ 10/1000 *μ*M/*μ*M, both combinations slightly suppressed cell proliferation, but did not affect cell viability (Figures 7(b) and 7(d)). Vitamin K1 (phylloquinone; applied alone) suppressed the proliferation of leukemic lymphocytes only at high concentrations (10 *μ*M and 20 *μ*M) (Figure 7(a)). Its combination with ascorbate did not show a synergistic antiproliferative effect (Figure 7(b)). The antiproliferative effect of vitamin K2 (menaquinone-4; applied alone) at high doses was less pronounced than that of vitamin K1 (Figure 7(c)). The combination vitamin K2/ascorbate also did not show synergistic effect on cell proliferation and viability (Figure 7(d)).

These data can explain, at least partially, the selective cytotoxicity of M/A on cancer cells, but not on normal cells. Normal cells can convert menadione to menaquinone (vitamin K2) via UBIAD1-catalyzed prenylation [92]. Downregulation of UbiA prenyltransferase domain-containing protein 1 (UBIAD1), also known as transitional epithelial response protein 1 (TERE1), is a hallmark of a majority of cancers [93]. Therefore, conversion of menadione to vitamin K2 will be strongly suppressed in cancer cells. The cytotoxicity of vitamins K1 and K2 against cancer cells has been reported to be one and two orders of magnitude lower, respectively, than that of menadione [94].

ESR study has demonstrated that only provitamin K3 produces superoxide in the presence of ascorbate under alkaline condition, while vitamin K2 is much less active and vitamin K1 is inactive as generators of superoxide in the presence of ascorbate [94]. Sasaki et al. has reported that provitamin K3 induces selective/distinct mitochondrial-mediated cytotoxicity in cancer cells via inhibition of mitochondrial DNA (mtDNA) polymerase-*γ*, while vitamins K1 and K2 do not affect mtDNA polymerase-*γ* activity and have a negligible effect on cell viability [95].

In addition, the different cytotoxic/cytostatic effects of vitamin K derivatives in combination with ascorbate can be explained by their different hydrophobicity and uptake and distribution in cancer cells.

### 3.4. Effects of Menadione/Ascorbate on Other Markers of Oxidative Stress and Mitochondrial Functionality

**Figure 8:**
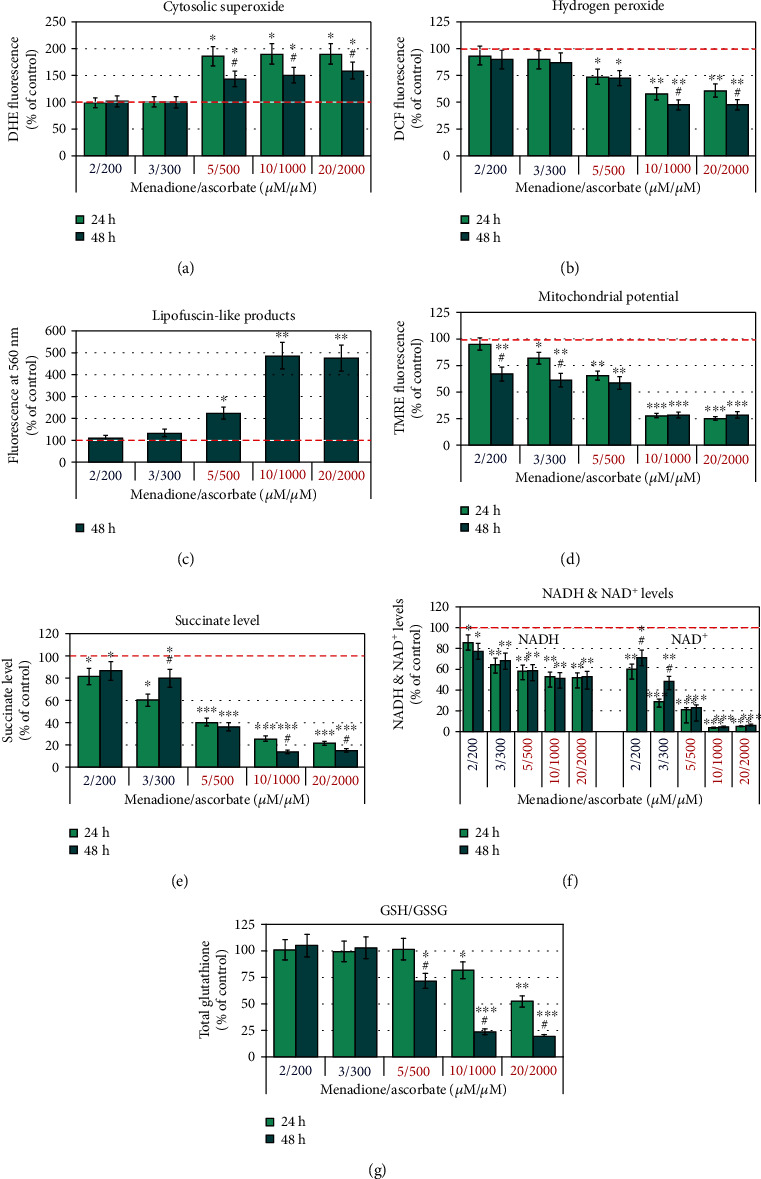
Effect of menadione/ascorbate on the steady-state levels of (a) “cytosolic” superoxide, (b) hydrogen peroxide, (c) lipofuscin-like products, (d) mitochondrial membrane potential, (e) succinate, (f) NADH and NAD^+^, and (g) total glutathione in leukemic lymphocytes, after 24 and 48 hours of incubation in humidified atmosphere. The number of cells in all samples at the beginning of each experiment was 1 × 10^6^ cells/mL. All data were normalized to 1 × 10^6^ cells and presented as a percentage of the respective control (untreated cells), which was considered 100%. The red dotted lines indicate the level of controls. The data are means ± SD from five independent experiments for (a) and three independent experiments for (b)–(g). In (c), fluorescence intensity at 560 nm was recorded at *λ*_ex_ = 490 nm. ∗*p* < 0.05, ∗∗*p* < 0.01, and ∗∗∗*p* < 0.001 versus the respective control (untreated cells). ^#^Statistically significant versus the respective M/A-treated cells for 24 hours.

To clarify the main source and general mechanism of oxidative stress in M/A-treated leukemic lymphocytes, we investigated the *steady-state levels of “cytosolic” superoxide, hydrogen peroxide, and end-products of free-radical oxidation* (Figures 8(a)–8(c)). The level of “cytosolic” superoxide was analyzed fluorimetrically using dihydroethidium (DHE) as a sensor. DHE is a MitoSOX analogue without triphenylphosphonium group. It is assumed that DHE is evenly distributed within the cytosol, as well as in all subcellular fractions [71]. The level of “cytosolic” superoxide was found to increase ~50-80% in leukemic lymphocytes treated with ≥5/500 *μ*M of M/A for 24 and 48 hours, but not in cells treated with low/tolerable M/A concentrations (Figure 8(a)). Hydrogen peroxide decreased ~25-50% in cells treated with ≥5/500 *μ*M of M/A, but it was at the control level in cells treated with low/tolerable concentrations of M/A (Figure 8(b)).

It should be noted that the M/A-induced changes of “cytosolic” superoxide and hydrogen peroxide were small compared to those observed for mitochondrial superoxide (Figures 8(a) and 8(b) versus Figure 4(c)).

A very good negative correlation was found between cell proliferation/viability and superoxide levels: *R* = –0.89 (*p* < 0.01) for mitochondrial superoxide and *R* = –0.90(*p* < 0.001) for “cytosolic” superoxide (Figure 3S in *Supplementary Materials*). This implies a decrease of cell viability with an increase of intracellular superoxide.

The correlation between cell proliferation/viability and hydrogen peroxide was positive as well (*R* = 0.85; *p* < 0.05) (Figure 3S in *Supplementary Materials*). It should be noted that the method detects the steady-state level of hydrogen peroxide, which depends on the balance between its production and consumption. M/A may induce abnormal generation of hydrogen peroxide in the extracellular fluids and inside the cells. However, hydrogen peroxide can also be degraded in Fenton's reactions, induced by high concentrations of M/A, as described in the literature [28, 30, 74, 79]. Studies in dynamics can help to clarify the exact mechanism and the root cause of M/A-induced severe oxidative stress in cancer cells. It should be noted that the level of DCF fluorescence, respectively, hydrogen peroxide, correlated positively with the level of glutathione in M/A-treated leukemic lymphocytes (*R* = 0.9974; *p* < 0.05) (Figure 3S in *Supplementary Materials*). The correlation between both parameters is described by Tampo et al. [96]. The authors have reported that glutathione depletion and aconitase activation are responsible for transferrin-mediated iron signaling in endothelial cells exposed to hydrogen peroxide and other hydroperoxides, which is accompanied by oxidation of DCFH to DCF.

End-products of oxidative stress, such as thiobarbituric acid-reactive substances, protein-carbonyl and exocyclic DNA adducts, and lipofuscin-like products, are the common markers for induction of Fenton/Haber-Weiss reactions in biological subjects [97]. We found that lipofuscin-like products increased significantly in leukemic lymphocytes, treated with ≥5/500 *μ*M of M/A for 48 hours (Figure 8(c)). Similar data on end-products of oxidative stress in cancer cells, treated with high concentrations of M/A, have been reported by other authors [28]. This explains, at least partially, the decrease in steady-state levels of hydrogen peroxide in cells treated with high concentrations of M/A, observed in our study (Figure 8(b)). It appears that degradation of hydrogen peroxide is faster than its production. However, at low/tolerable concentrations of M/A, lipofuscin-like products and hydrogen peroxide were almost at their control levels found in untreated cells.

As mentioned in *Introduction*, the anticancer effect of M/A has been attributed by other authors to abnormal generation of hydrogen peroxide in the cytosol and extracellular space and subsequent induction of severe oxidative stress via Fenton/Haber-Weiss reactions [28, 30, 79, 80]. These assumptions are based on indirect evidence: (i) decrease of M/A-mediated cytotoxicity by metal chelators and (ii) accumulation of end-products of free radical oxidation in M/A-treated cells. The data about the effect of metal chelators on M/A-treated cells are disputable, considering the experimental conditions used. Induction of Fenton's reactions and accumulation of end-products of free radical oxidation in M/A-treated cancer cells are attributed to the presence of free iron in these cells, as well as in the extracellular matrix of solid tumors. Iron ions can decompose hydrogen peroxide to hydroxyl radicals, which leads to induction of free radical oxidation and destruction of biomembranes and biomacromolecules. However, this mechanism for production/consumption of hydrogen peroxide is unlikely given that metalloproteins (such as ferritin) are usually overexpressed in cancer cells [98] and iron ions should therefore be complexed. Most likely, it is just an accompanying pathway, which occurs at high concentrations of M/A, but not at low/tolerable concentrations of this combination drug.

Overproduction of mitochondrial superoxide in M/A-treated leukemic cells (Figure 4(c)) can explain, at least partially, the decrease of steady-state levels of hydrogen peroxide at high doses (Figure 8(b)). It is well known that high concentrations of superoxide can attack Fe-S clusters in mitochondrial complexes and induce the release of free Fe ions [99, 100]. In turn, Fe ions can decompose hydrogen peroxide through Fenton's reactions and ascorbate accelerates this process [17, 97]. This mechanism is tightly connected to the cancerous mitochondria and should be specific for M/A-treated cancer cells, but not for normal cells of the same origin. In additional experiments, we found that ferroptosis inhibitor (ferostatin-1) and necrosis inhibitor (necrostatin-1) did not abolish the cytotoxic/cytostatic effect of M/A on leukemic lymphocytes up to 5/500 *μ*M/*μ*M of M/A, but slightly abolished the cytotoxicity of high concentrations of M/A (Figure 4S in *Supplementary Materials*). These data suggest that both mechanisms of cancer cell death are not dominant for M/A-treated cancer cells, particularly for leukemic lymphocyte Jurkat. In the course of the experiments, using light microscopy and automatic counting to normalize cell suspensions by equal number, we noticed that the size of M/A-treated cancer cells was significantly reduced compared to that of untreated cells, even at low/tolerable concentrations of M/A. This observation is too close to the described new form of cell death “autoschizis” [59–65], although we found a significant expression of PSer on the cell surface of M/A-treated leukemic lymphocytes—a conventional marker for induction of apoptotic cell death (please, see subsection 3.6 below).

Generation of superoxide and hydrogen peroxide has been shown for a number of 1,4-naphthoquinones applied separately or in combination with ascorbate in model cell-free systems [30, 101, 102]. Studies demonstrate a production of superoxide and hydrogen peroxide in cells treated with high concentrations of menadione or ascorbate, applied separately [18, 103]. However, there are no articles demonstrating M/A-induced overproduction of superoxide in cells. *Based on our knowledge, the present study is the first to show that M/A induces much higher superoxide production in cancer cells than in normal cells, and cancerous mitochondria are the major source. The mitochondrial redox cycling of menadione and ascorbate appears to underlie this process and is likely to be different in normal and cancer cells.*

*Depolarization of the mitochondrial membrane* of leukemic lymphocytes with increasing M/A concentration (Figure 8(d)) is further evidence that mitochondria are the target of this combination. Mitochondrial potential of M/A-treated cancer cells decreased to more normal values even at low/tolerable concentrations.

*Succinate level* also decreased with increasing M/A concentration in leukemic lymphocytes (Figure 8(e)). The decrease of succinate is very well observable at 24 hours of incubation. At 48 hours of incubation with 2/200 and 3/300 *μ*M/*μ*M of M/A, the succinate levels begin to normalize (similar to the effect on ATP; Figure 3(b)), but do not reach the initial control level. At high concentrations of M/A (>5/500 *μ*M/*μ*M), succinate decreased with incubation time. Succinate is considered one of the major oncometabolites [104, 105]. It was found that Krebs cycle metabolites (such as succinate, fumarate, and itaconate) are coupled with nonmetabolic signaling in cancer and immune cells, which is crucial for cancer progression and invasion [106, 107]. Therefore, suppression of succinate production in cancer cells and modulation of the immune response may underlie the anticancer effect of relatively low/tolerable doses of M/A in vivo, reported in the literature. Decrease of succinate can also explain the anti-inflammatory effect of M/A, described recently [108].

*NADH and NAD^+^* also gradually decreased in leukemic lymphocytes depending on the concentration of M/A (Figure 8(f)). NADH depletion was moderate, but irreversible within 48 hours of incubation. NAD^+^ depletion was very strong, but reversible for the time of incubation, especially at low/tolerable concentrations of M/A. Depletion of NAD^+^ in cancer cells treated with high concentrations of M/A (>5/500 *μ*M/*μ*M) is described by several groups [4, 24–26, 109]. The authors have explained this phenomenon by the activation of poly-[ADP ribose] polymerase 1 (PARP1), inhibition of glycolysis, subsequent ATP depletion, and cell death. This mechanism is attributed to the oxidative stress, induced by menadione/ascorbate. It should be noted that the mitochondrial NAD “pool” is not directly connected to the cytoplasmic NAD “pool” and their data cannot be extrapolated to mitochondrial functionality. It is not surprising that high concentration of M/A decreases the glycolytic rate, because (i) ascorbate competes with glucose for the GLUT1 transporter [67, 87], ascorbate concentration of 2000 *μ*M is comparable to, and competes with, glucose concentrations used in the culture media, and (ii) inhibition of glyceraldehyde phosphate dehydrogenase as a result of oxidative stress at high doses of M/A [110, 111]. The mechanism of M/A-mediated activation of PARPs and severe depletion of NAD^+^ and ATP seems probable at high doses (>5/500 *μ*M/*μ*M). The partial depletion and recovery of NAD^+^ with the incubation time at low/tolerable concentrations of M/A (Figure 8(f)) indicate the reversibility of this process and its limited contribution in the overall anticancer effect at doses, attainable in cancer in vivo by oral and parenteral administration.

The molecular mechanism(s) of anticancer and anti-inflammatory effects of low/tolerable doses of M/A, achievable with oral administration, based on the redox cycling of both compounds and induction of oxidative stress, seems much more complex than mere activation of PARPs and subsequent ATP depletion, leading to cell death.

### 3.5. Role of NQO1 for Menadione/Ascorbate-Induced Cytotoxicity and Overproduction of Mitochondrial Superoxide in Cancer Cells

One of the key intracellular regulators of the redox cycling of menadione is NQO1 [30, 34–38], which is overexpressed in various cancers [39, 40].

**Figure 9:**
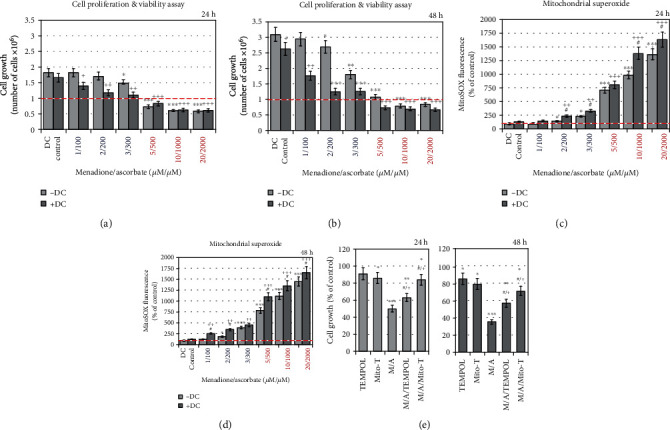
(a, b) (DC; 25 *μ*M) on proliferation activity of menadione/ascorbate- (M/A-) treated leukemic lymphocytes, after 24 and 48 hours of incubation, analyzed by trypan blue staining and automated cell counting. The red dotted line indicates the initial number of cells. (c, d) Effect of dicoumarol on the level of mitochondrial superoxide in menadione/ascorbate-treated leukemic lymphocytes, after 24 and 48 hours of incubation, analyzed by MitoSOX fluorescence. The red dotted line indicates the level of MitoSOX fluorescence in the control (nontreated cells), which was considered 100%. (e) Effect of mito-TEMPO (mito-T; 100 *μ*M) and TEMPOL (500 *μ*M) on proliferation activity of M/A-treated leukemic lymphocytes, analyzed by trypan blue staining and automated cell counting after 24 and 48 hours of incubation. Concentration of M/A was 5/500 *μ*M/*μ*M. The proliferation activity of the treated cells was presented as a percentage of the proliferation activity of control (untreated cells), which was considered 100%. The number of cells in all samples at the beginning of each experiment was 1 × 10^6^ cells/mL. The data are means ± SD from fifteen independent experiments for (a) and (b) in the absence of dicoumarol, three independent experiments for (a) and (b) in the presence of dicoumarol, and four independent experiments for (c)–(e). ∗*p* < 0.05, ∗∗*p* < 0.01, and ∗∗∗*p* < 0.001 versus the respective control group (untreated cells or cells treated with DC only). In (a)–(d): ^#^*p* < 0.05, comparison between the respective untreated and DC-treated cells in the presence of M/A. In (e): ^#^*p* < 0.05, comparison between the respective nitroxide-treated cells in the absence and presence of M/A. ^+^*p* < 0.05, comparison between the nitroxide/M/A-treated cells versus M/A-treated cells only.

We investigated the role of NQO1 for the M/A-induced cytotoxicity and overproduction of mitochondrial superoxide in leukemic lymphocytes, using dicoumarol, a selective NQO1 inhibitor (Figures 9(a)–9(d)). In the absence of M/A, dicoumarol (25 *μ*M) had no effect on cell viability, but slightly suppressed cell proliferation (~10-15% for 48 hours) (Figures 9(a) and 9(b)). The drug potentiated the antiproliferative effect of low/tolerable concentrations of M/A, even at 1/100 *μ*M/*μ*M. It increased the cytotoxicity of high concentrations of M/A at 48 hours of incubation (Figure 9(b)).

In the absence of M/A, dicoumarol slightly increased mitochondrial superoxide (~20-30% above the control level) (Figures 9(c) and 9(d)). Dicoumarol potentiated the effect of M/A on the level of mitochondrial superoxide; it increases from ~20% to 2 times (depending on M/A concentration) compared to M/A-treated leukemic lymphocytes in the absence of dicoumarol. The effect of dicoumarol was better expressed at low concentrations of M/A (1/100 and 2/200 *μ*M/*μ*M).

Recently, Glorieux and Calderon have reported that NQO1 contributes to the cytotoxicity of M/A [109]. The authors have investigated the sensitivity of cell lines with different expression and activity of NQO1. Their data suggest that cell sensitivity to M/A correlates with NQO1 activity—the cells with the highest activity are more resistant to M/A. In this context, NQO1 activity can be used as a marker for predicting the effect of M/A on cancer cells, as well as for stratifying patients with potential response to M/A in adjuvant settings of cancer therapy.

In contrast, substances (such as cyclic nitroxides) that decrease the level of superoxide abolished the cytotoxicity of M/A in leukemic lymphocytes (Figure 9(e)). We used two superoxide scavengers: (i) TEMPOL, which is evenly distributed inside the cells, and (ii) mito-TEMPO, which is localized in the mitochondria [71, 112]. At the selected experimental conditions, mito-TEMPO completely abolished M/A-mediated cytotoxicity on leukemic cells, while the effect of TEMPOL was less pronounced (Figure 9(e)).

### 3.6. Sensitizing Cancer Cells to Conventional Anticancer Drugs Using Low/Tolerable Doses of Menadione/Ascorbate

**Figure 10:**
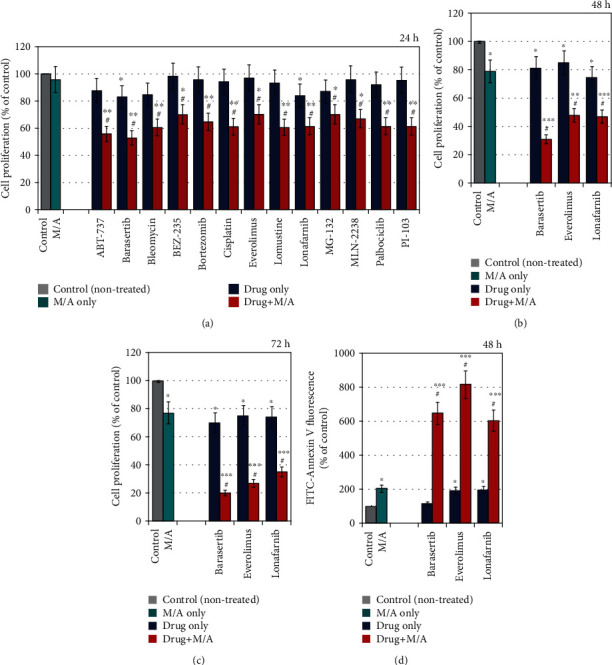
(a) Effects of menadione/ascorbate (M/A) and anticancer drugs applied separately and in combination on proliferation activity of leukemic lymphocytes, after 24 hours of incubation in humidified atmosphere, analyzed by trypan blue staining and automated cell counting. The proliferation activity of the treated cells was presented as a percentage of the proliferation activity of control (untreated cells), which was considered 100%. The number of cells in all samples at the beginning of each experiment was 1 × 10^6^ cells/mL. The data are means ± SD from six independent experiments. (b, c) Synergistic antiproliferative effect of M/A in combination with barasertib, everolimus, and lonafarnib at 48 and 72 hours of incubation. (d) Induction of apoptosis in leukemic lymphocytes treated with M/A alone and in combination with barasertib, everolimus, and lonafarnib for 48 hours. Drug concentrations: menadione/ascorbate: 3/300 *μ*M/*μ*M, ABT-737: 0.1 *μ*M, barasertib: 0.05 *μ*M, bleomycin: 0.5 *μ*M, BEZ-235: 0.025 *μ*M, bortezomib: 0.01 *μ*M, cisplatin: 2.5 *μ*M, everolimus: 5 *μ*M, lomustine: 10 *μ*M, lonafarnib: 0.5 *μ*M, MG-132: 0.025 *μ*M, MLN-2238: 0.01 *μ*M, palbociclib: 0.25 *μ*M, and PI-103: 0.5 *μ*M. ∗*p* < 0.05, ∗∗*p* < 0.01, and ∗∗∗*p* < 0.001 versus the respective control group. ^#^Statistically significant versus drug-treated cells only.

In the present study, we also investigate the possibility of low/tolerable concentration of M/A (3/300 *μ*M/*μ*M) to sensitize leukemic lymphocytes to conventional chemotherapeutics that are used to treat leukemia (Figure 10). Eighteen anticancer drugs were analyzed. Thirteen of them showed a synergistic antiproliferative effect after 24 hours of incubation with M/A (Figure 10(a)). For three drugs (barasertib, everolimus, and lonafarnib), synergism increased with the incubation time (Figures 10(b) and 10(c)), which was accompanied by a very strong induction of apoptosis (Figure 10(d)). For the combinations of M/A with the other 10 drugs, the effect became additive over time. It can be speculated that the sensitizing properties of M/A are due to overproduction of mitochondrial superoxide and impairment of cancer mitochondria. This can increase the sensitivity of cancer cells to conventional chemo- and radiation therapy. Impairment of mitochondrial respiration by M/A could also affect the NAD(P)^+^/NAD(P)H ratio and succinate level (Figures 8(e) and 8(f)) and thus could influence the native immune response against cancer in vivo even at low/tolerable doses. It has been demonstrated that M/A suppresses the activities of matrix metalloproteinases and urokinase plasminogen activator (uPA) and thus inhibits tumor growth and exhibits antimetastatic potential [25, 52]. M/A also sensitizes cancer cells to natural killer cells [113].

### 3.7. Effect of M/A on Tumor Growth in Cancer-Bearing Mice and PD-L1 Expression on Cancer Cells

**Figure 11:**
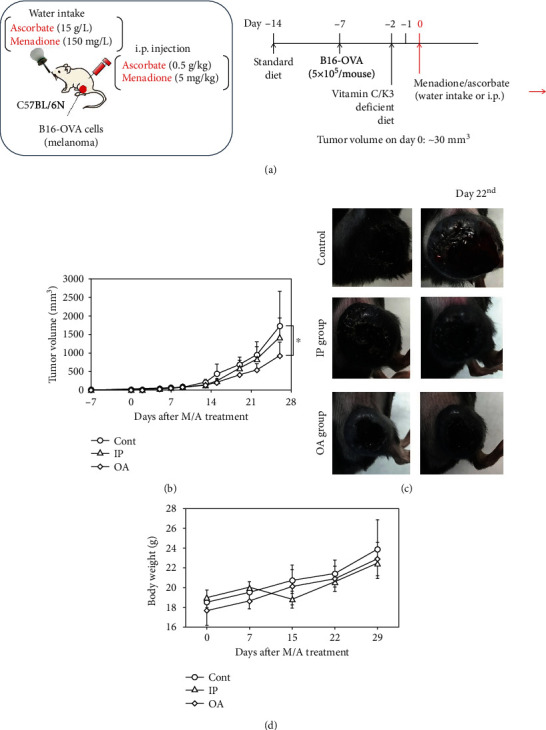
(a) Experimental design in vivo. (b) Antitumor effect induced by intraperitoneal (IP) or oral administration (OA) of menadione/ascorbate (M/A) in melanoma-bearing C57Bl/6N mice, estimated by tumor volume. (c) Representative photos of tumors in mice obtained on day 22 after starting M/A administration. (d) Effect of M/A on body weight of melanoma-bearing C57Bl/6N mice. In (b) and (d), the data are means ± SD from five mice in each group. ^∗^Statistical significance on day 22 (*p* < 0.05).

Oral administration of M/A suppressed significantly the tumor growth compared to the control group (Figures 11(b) and 11(c)). Intraperitoneal administration did not show statistically significant difference compared to the control group until day 29 from the start of the treatment. This is difficult to explain, given that intraperitoneal treatment should lead to higher concentrations of M/A in plasma, as well as cells and tissues, including the tumors. We believe this is indirect evidence that low/tolerable doses of M/A have a stronger anticancer effect in vivo than high pharmacological doses. M/A did not affect significantly mouse body weight (Figure 11(d)).

Our hypothesis is that M/A could affect the immune system and increase the recognition of cancer cells and their partial elimination, respectively. Our current efforts are directed to prove or deny this hypothesis.

**Figure 12:**
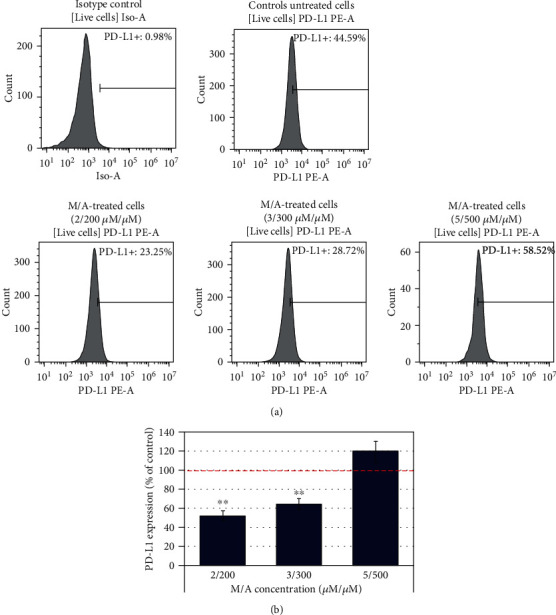
FACS analysis of PD-L1 expression in B16-F10 melanoma cells treated with menadione/ascorbate (M/A) for 72 hours. (a) Representative FACS histograms. (b) PD-L1 expression in live M/A-treated cells as a percentage of control untreated cells. In the chart, the data are means ± SD from two independent experiments. ∗∗*p* < 0.01 versus the control (untreated cells). Note: this analysis was conducted (commercially) by Crown Bio Inc., Beijing, China.

In an experimental model in vitro (pilot study), using the same melanoma cell line B16-F10, we investigated the effect of M/A on the expression of programmed cell death ligand 1 (PD-L1). PD-L1 is a transmembrane protein that is overexpressed in cancer cells and plays a major role in suppressing the adaptive immune response (in particular, T lymphocytes) [114, 115]. The PD-1/PD-L1 checkpoint pathway is one of the most promising targets of modern cancer immunotherapy [116, 117]. Our data demonstrate that low/tolerable concentrations of M/A, achievable by oral administration, significantly suppressed PD-L1 expression on melanoma B16-F10 cells, while high concentrations of M/A (5/500 *μ*M/*μ*M) had no effect on this parameter (Figure 12). Similar effects have been also obtained on other cancer cell lines (data will be published elsewhere). These data suggest that M/A may have a beneficial effect on the immune system of the cancer-bearing organism, making the cancer cells “visible” and perhaps more vulnerable to native immune cells.

Other studies on experimental animals have reported that oral and parenteral M/A potentiates the efficiency of conventional chemotherapy and radiotherapy of cancer in vivo and inhibits invasion and metastasis [46–48]. The weights of the spleen and thymus are higher in M/A-treated animals compared with those receiving conventional drugs alone [46–48]. This suggests involvement of mechanisms related to immune stimulation that have to be explored. However, no studies on the effect of M/A on the immune response have yet been published. Other authors have established that M/A (1 g/kg ascorbate and 10 mg/kg menadione sodium bisulfite (MSB); i.p. administration) significantly increased the life span of TLT-bearing C3H mice, as well as potentiating or sensitizing cancer to conventional chemotherapeutics such as cyclophosphamide and Oncovin® [1, 18, 46, 47, 50]. Neither menadione nor ascorbate, when administered separately, affected the life span of TLT-bearing animals. A significant decrease of lung metastases has also been reported in this cancer model in vivo, after oral administration of M/A (15 g/L ascorbate and 150 mg/L MSB in drinking water) and X-ray exposure (20 Gy) [1, 48, 50]. These studies have demonstrated that oral *ad libitum* administration of clinically attainable doses of M/A in drinking water did not produce any adverse side-effects in mice, or macroscopic/microscopic pathological alterations in normal tissues. Kassouf et al. have reported that M/A substantially augmented the anticancer effect of gemcitabine in vivo [51]. The combination of gemcitabine and M/A significantly reduces the tumor growth in nude mice inoculated with metastatic human urothelial carcinoma cells, compared to gemcitabine administered alone. The authors conclude that M/A enhances the sensitivity of cancer to gemcitabine and may allow application of lower, less toxic doses of this anticancer drug in second-line therapy. Beck et al. have observed that M/A (1 g/kg ascorbate and 10 mg/kg MSB; i.p. or i.v. administration) markedly suppresses tumor growth and increases survival of Balb/c nude mice with leukemia and myeloma [21]. Chen et al. have examined the effects of M/A in low dose (100 mg/kg ascorbate and 1 mg/kg menadione) and high dose (1 g/kg ascorbate and 10 mg/kg menadione), administered i.p. in LLC-bearing C57Bl/6 mice [52]. These authors observed that M/A inhibits tumor growth and metastatic potential in vivo in a dose-dependent manner, and the effect of high-dose M/A is comparable to cisplatin at 6 mg/kg in LLC-bearing mice. However, cisplatin-treated animals had significant weight loss, whereas cisplatin/M/A-treated animals maintained the body weight at the level of healthy mice.

## 4. Discussion

Summarizing, our study demonstrates that the combination of menadione and ascorbate exhibits a highly specific and synergistic suppression on cancer cell growth and viability, without adversely affecting the viability of normal cells at pharmacologically achievable concentrations. This targeted anticancer effect of M/A is the result of a selective redox cycling between both substances within dysfunctional mitochondria.

The cytostatic/cytotoxic effect of M/A in cancer cells is accompanied by

- an extremely high production of mitochondrial superoxide in cancer cells, but not in normal cells of the same origin, after their treatment with M/A. This effect is manifested even at 2/200 *μ*M/*μ*M of M/A (for 48 hours of incubation), which is attainable by oral and parenteral administration of both ingredients [1, 67]. It is M/A-specific and is not well expressed by the administration of menadione or ascorbate alone
- a significant time-dependent decrease of mitochondrial membrane potential even at low/tolerable concentrations of M/A
- a slight decrease of the steady-state levels of ATP in cancer cells, treated with low/tolerable concentrations of M/A for 24 hours, and recovery of the ATP to the control level after 48 hours of treatment
- succinate depletion in M/A-treated cancer cells (concentration dependent). The effect is reversible over time at low/tolerable doses, but irreversible at high doses
- NADH and NAD^+^ depletion in M/A-treated cancer cells (concentration dependent). The effect on NAD^+^ is reversible over time at low/tolerable doses, but irreversible at high doses

In addition, upon treatment of leukemic lymphocytes with high concentrations of M/A, we found a relatively slight increase of “cytosolic” superoxide, decrease of hydrogen peroxide, and increase of end-products of oxidative stress within 48 hours. These parameters were at control levels after treatment with low/tolerable concentrations. Inhibition of NQO1 by dicoumarol increased mitochondrial superoxide and sensitized leukemic lymphocytes to M/A. The combination of ascorbate with menadione exhibited a synergistic anticancer effect, which was not observed when ascorbate was combined with vitamins K1 or K2.

These data demonstrate a severe mitochondrial oxidative stress in M/A-treated cancer cells, which is concentration dependent. Such effect is not observed in normal cells of the same origin. M/A-treated normal cells are characterized by induction of mild oxidative stress, which seems to be tolerated. The data also suggest that superoxide/hydrogen peroxide production dominates within the mitochondria of M/A-treated cancer cells, while their utilization dominates in the cytosol, especially at high concentrations of M/A.

**Figure 13:**
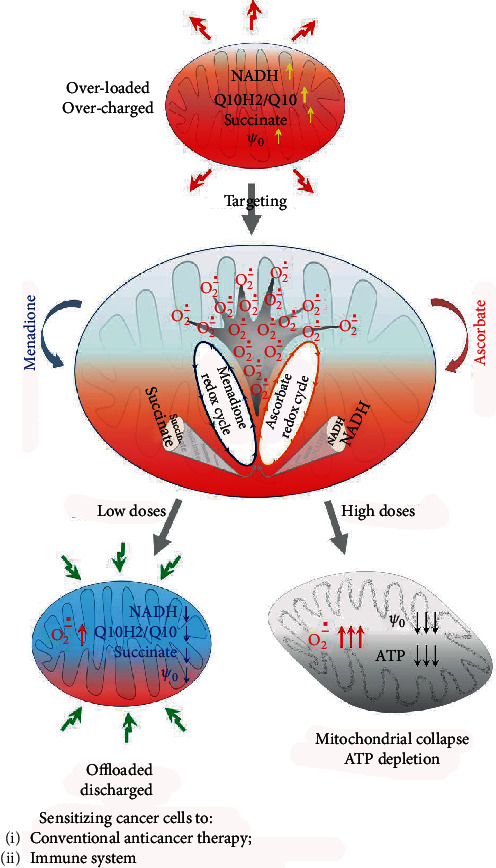
Schematic representation of mechanism of M/A-mediated effect on cancerous mitochondria. Cancer cells are overloaded with reducing equivalents, such as NADH and succinate, as well as overcharged due to the high Q10H2/Q10 ratio [68, 104–107, 118, 119]. Menadione/ascorbate (M/A) decreases mitochondrial membrane potential and increases the level of mitochondrial superoxide (found in our study). Overproduction of superoxide requires electrons, coming from reducing equivalents. This leads to depletion of succinate and NADH in mitochondria (found in our study). At high doses of M/A, these processes lead to mitochondrial collapse and decrease of cell viability. At low/tolerable doses of M/A, these processes could lead to increasing sensitivity of cancer cells to conventional anticancer drugs, radiation therapy, and immune system.

Possible reasons for the specific overproduction of mitochondrial superoxide in M/A-treated cancer cells are (i) a direct impairment of mitochondrial ETC by compromising its functionality (mainly complex I and complex III that are known to produce superoxide [118, 119]) and (ii) a specific mitochondrial redox cycling of both substances, mediated by dysfunctional mitochondria, but not by the mitochondria of nontransformed cells (Figure 13).

We also observed a significant irreversible ATP depletion in cancer cells treated with ≥5/500 *μ*M/*μ*M of M/A for 24 and 48 hours, but not with 2/200 and 3/300 *μ*M/*μ*M of M/A (Figure 3(b)). Therefore, direct irreversible ETC damage appears to be inherent to high concentrations of M/A (≥5/500 *μ*M/*μ*M), but not in low/tolerable concentrations. It is interesting to note that low/tolerable concentrations of M/A induce superoxide production and decrease mitochondrial potential in cancer cells, especially after 48 hours of incubation (Figures 4(c) and 8(d)), although they do not affect the level of ATP. This implies a significant interference with mitochondrial respiration, even at low/tolerable concentrations of M/A. Overproduction of superoxide requires electrons and should be tightly connected to consumption of reducing equivalents in mitochondria. Depletion of NADH and succinate in M/A-treated cancer cells confirms this assumption (Figures 8(e) and 8(f)).

Currently, we cannot answer the question “Whether or not and how M/A induces direct inhibition of ATP synthesis in cancerous mitochondria at high concentrations.” However, abnormal concentration of mitochondrial superoxide in M/A-treated cells definitely is a factor disrupting the ETC and ATP synthesis. In this context, the depletion of ATP observed in cancer cells at high concentrations of M/A could be a consequence of two processes: inhibited glycolysis and impaired mitochondrial function.

Our data show some important additional findings: (i) mitochondrial superoxide increases linearly with increasing M/A concentration in cancer cells, but not in normal cells (Figures 4(c), 5(b), and 5(e)); (ii) overproduction of superoxide in cancerous mitochondria does not depend on the level of ATP (Figures 4(c), 5(d), and 3(b)); (iii) there is no difference in the effects of ascorbate/menadione and ascorbate/menadiol on cell proliferation and viability (Figures 6(c) and 6(d), solid columns); (iv) dehydroascorbate in combination with menadione or menadiol manifests similar cytostatic/cytotoxic effect as in the case of ascorbate (Figures 6(c), 6(d), dashed columns). These results point to the existence of a specific mechanism(s) for M/A-mediated superoxide production in cancerous mitochondria. We suspect this is most likely due to

- a permanent and specific enzyme-supported and/or non-enzyme (succinate)-supported redox cycling between menadiol/menadione in the mitochondria
- a one-electron redox cycling between menadione and ascorbate as a result of specific appearance of unprenylated menadione in dysfunctional mitochondria only

A specific mitochondrial redox cycling of menadione/menadiol is described in the study of Chan et al. [14]. It occurs between NQO1 and complex III of the inner mitochondrial membrane and leads to conversion of menadiol to menadione. Thus, menadione (below 5 *μ*M) could bypass complex I and increase ATP production in complex I-deficient cells. The same mechanism is shown for coenzyme Q0 (CoQ0), a precursor of CoQ10 [14]. We also observed that 5/500 *μ*M/*μ*M of M/A is a crucial concentration for the recovery of ATP, NAD^+^, and succinate levels in M/A-treated cancer cells (Figure 3(b)). Excess menadione, produced by complex III only in mitochondria, may undergo one-electron redox cycling at this locus, increasing superoxide production. This is accelerated by ascorbate or other one-electron redox cycling mechanisms, as described in *Introduction* (Figures 1(a) and 1(b)).

The effect of the NQO1 inhibitor dicoumarol on M/A-mediated mitochondrial superoxide production and suppression of proliferation (Figure 9) also suggests that redox cycling of menadione contributes to these effects. Several forgotten studies from the 1980s have reported the so-called “antimycin A-independent” and “cyanide-dependent” oxygen consumption, induced by menadione on isolated mitochondria and cell and tissue homogenates [120, 121]. The authors have described an oxygen consumption that comes from specific mitochondrial redox cycling of menadione. Recent studies also support this assumption [122–124]. The authors have described a mitochondrial redox cycling of menadione and its mitoquinone derivative, which is accompanied by abnormal production of superoxide, induction of apoptosis, and anti-inflammatory and antifibrotic effects. These studies give a reason to suspect that mitochondrial redox cycling of menadiol back to menadione could adversely affect cancer cell metabolism. This mechanism is very likely to underlie the specific anticancer effect of M/A, as well as its ability to sensitize cancer cells to conventional anticancer drugs (Figure 10).

It is well known that cancer cells produce high levels of succinate, the second source for ATP synthesis after NADH [104–107]. Succinate is well recognized as an oncometabolite, which increases due to increased glutaminolysis and impaired Krebs cycle in cancer cells and tissues. More recent studies on cancer cell metabolism also show a very high ratio of NADH/NAD^+^ in cancer due to upregulation of the aldehyde dehydrogenase pathway [68, 125]. This phenomenon of overcharging of cancerous mitochondria with primary reducing equivalents (NADH and succinate) should be tightly connected to the redox potential of mitochondrial “Q-pools”. High amounts of NADH and succinate cannot exist unless the “Q pool” is overreduced. This is a very important biochemical/physiological phenomenon, distinguishing cancer cells from normal cells [126]. Studies on ischemia-reperfusion injury (IRI) have demonstrated that the phenomenon of overcharged “Q-pools” leads to overproduction of superoxide by complex I and complex III [127]. The superoxide obtained by complex III is associated with the appearance of Q10 semiquinone [128]. Compared with Q10, menadione and menadiol are more mobile small molecules due to the lack of a long hydrophobic tail. This makes them more available electron carriers than long-tailed Q10. Their interference with the active sites of ETC complexes could result in the production of (i) much more semiquinone from menadione than from Q10 and (ii) as a consequence more superoxide.

High concentrations of ascorbate (≥1000 *μ*M), applied alone, also increased the level of mitochondrial superoxide (Figure 4(a)). It is assumed that ascorbate may directly affect mitochondrial respiration in cancer cells by transferring electrons to cytochrome *c* and thus blocking electron transfer from complex III [118, 119, 129–131]. Mitochondrial redox cycling of menadione and ascorbate can explain, at least partially, the synergistic effect of M/A on the level of mitochondrial superoxide and proliferation activity of cancer cells.

Our hypothesis is that menadione and ascorbate are able to discharge/offload cancerous mitochondria from the overreduced state, by their mitochondrial redox cycling (Figure 13). The effect is best expressed when the two substances are combined to cause synergistic overproduction of superoxide within dysfunctional mitochondria. Once superoxide exceeds a certain threshold, vital functional mitochondrial units (such as ETC and ATP synthase) can be destroyed and cause inhibition of mitochondrial ATP synthesis, leading to the release of caspase-independent apoptosis-inducing factor (AIF) and cell death [132, 133].

It is generally accepted that cancer cells use glycolysis as a source of ATP and do not rely on mitochondrial respiration for ATP production. However, recent studies demonstrate that the majority of ATP in cancer cells is produced by mitochondria and some tumors show heavy dependence on oxidative phosphorylation [134–136]. Many solid tumors are poorly perfused and have a limited supply of glucose, but enough oxygen to generate mitochondrial ATP [137]. The ETC is able to function optimally at oxygen levels as low as 0.5% [138]. Therefore, blocking mitochondrial respiration will induce cell death in poorly perfused tumors. Even ignoring the dependence of cancer cells on mitochondrial ATP, a functioning of Krebs cycle (at complex II), is vital for the supply of metabolites for synthesis of nucleic acids, fatty acids, etc. [106, 107]. Blocking the ETC will stop this supply and suppress proliferation also by inhibiting the orotate dehydrogenase and pyrimidine synthesis [139, 140]. Krebs cycle metabolites (such as succinate, fumarate, and itaconate) are also coupled with nonmetabolic signaling in cancer and immune cells, which is crucial for cancer progression and invasion [106, 107].

We assume that the ability of normal cells to convert menadione to vitamin K2 (which has a membrane binding tail) is diminished or absent for cancer cells due to the downregulation of UBIAD1 (TERE1) [92, 93]. Combined with relatively low intracellular levels of ascorbate and balanced “Q-pools” within properly functioning mitochondria [67, 126], this set of favorable factors protects nontransformed cells from the harmful effects of the M/A.

## 5. Conclusion

The studies published in the literature suggest two mechanisms of anticancer effects of menadione/ascorbate: (i) extracellular generation of hydrogen peroxide due to ascorbate/menadione one-electron redox cycling and subsequent induction of oxidative stress, accompanied by activation of PARP1, inhibition of glycolysis, depletion of NAD^+^ and ATP, and subsequent cell death [4, 24–26, 110, 111] and (ii) intracellular (“cytosolic”) generation of hydrogen peroxide due to ascorbate/menadione redox cycling and severe oxidative and replicative stress due to induction of Fenton's reactions [28, 30, 79].

However, both mechanisms are nonspecific (general) and cannot explain (i) why M/A attacks cancer cells but not normal cells and (ii) why the anticancer effects of M/A in vivo are demonstrated at significantly lower plasma concentrations than those inducing cancer cell death in vitro [1, 3]. Cancer cells have a variety of mechanisms to control oxidative stress and survive, and they are much more resistant than normal cells [68, 69]. Direct evidence of this are (i) the control steady-state levels of cytosolic superoxide, hydrogen peroxides, and lipofuscin-like products and (ii) the recovery of ATP and NAD^+^ levels with longer times of treatment, in cancer cells treated with low/tolerable doses of M/A (Figures 3(b) and 8). Our data demonstrate that M/A induces a specific overproduction of mitochondrial superoxide and severe oxidative stress in cancer cells, which is concentration dependent and time dependent. In normal cells of the same origin, M/A induces a relatively low increase of mitochondrial superoxide in a concentration-independent manner and mild, well-tolerated oxidative stress, which seems to be controlled (Figures 4(c) and 5). Most likely, this modest oxidative stress is a result of extracellular and cytosolic redox cycling of menadione and ascorbate with production of hydrogen peroxide. The similar effects of ascorbate and dehydroascorbate in combination with menadione or menadiol (Figures 6(c) and 6(d)) raise questions about the role of extramitochondrial hydrogen peroxide as a “primary trigger” of oxidative stress in cancer cells.

We observed that cancer cells, particularly leukemic lymphocytes (Jurkat), treated with low/moderate concentrations of M/A, are also characterized by (i) decreased mitochondrial potential at 48 hours of incubation, (ii) NADH and succinate remain below the control level, and (iii) gradual time-dependent increase of mitochondrial superoxide (Figures 4(c), 5(e), and 8(d)–8(f)). These findings are prerequisites for sensitizing cancer cells to conventional anticancer drugs (Figure 10), as well as enhancing their vulnerability to the immune system (Figure 13). This makes M/A a valuable “tool” in adjuvant settings of cancer therapy.

We propose that the anticancer effect of M/A is a result of a specific mechanism that is tightly connected to the cancerous mitochondria. This could be the result of “specific” mitochondrial redox cycling of menadione and ascorbate due to their inherent dysfunction. This hypothesis could be verified using Seahorse technology for real-time measurement of oxygen consumption rate using inhibitors of each ETC complex [141], which is the goal of our future studies.

Experimental data demonstrate that low/tolerable doses of M/A have no destructive effects on cancer cells, but they induce metabolic changes in them as (i) a decrease of succinate and NADH, (ii) a depolarization of mitochondrial membrane, and (iii) a specific overproduction of superoxide and severe oxidative stress in cancerous mitochondria only. In addition, low/tolerable doses of M/A suppress tumor growth in vivo after oral administration and downregulate PD-L1 in cancer cells (Figures 11 and 12). These results suggest a great potential of M/A for beneficial anticancer effects, as well as increased sensitivity and vulnerability of cancer cells to the conventional therapies, as well as to the immune system.

## Data Availability

Data in the supporting information are available on request via the following e-mail: bakalova.rumiana@qst.go.jp (Dr. Rumiana Bakalova).

## Supplementary Materials

Supplementary Materials Appendix 1: Full systematic study is being presented. Figure 1S: nitroxide redox cycle c EPR/MRI imaging of cellular redox status: principle of the method. Figure 2S: effects of catalase (150 *μ*g/mL or ~500-750 U/mL) on proliferation activity of M/A-treated leukemic lymphocytes, analyzed by trypan blue staining and automated cell counting. Figure 3S: correlation analysis between cell proliferation/viability and ROS of menadione/ascorbate-treated cells, described in Figures 2, 4, and 8 (two-sample equal variance). Figure 4S: effect of ferrostatin (Fer-1; 2 *μ*M) and necrostatin (Nec-1; 20 *μ*M) on proliferation activity of M/A-treated leukemic lymphocytes (Jurkat), analyzed by trypan blue staining and automated cell counting.
