# Supplementary material for: Selective Targeting of Cancerous Mitochondria and Suppression of Tumor Growth Using Redox-Active Treatment Adjuvant
Source: Oxid Med Cell Longev. 2020 Nov 2;2020:6212935. doi: 10.1155/2020/6212935 (PMC7652615; doi:10.1155/2020/6212935)
Supplement: Supplementary Materials — Appendix 1: Full systematic study is being presented. Figure 1S: nitroxide redox cycle c EPR/MRI imaging of cellular redox status: principle of the method. Figure 2S: effects of catalase (150 μg/mL or ~500-750 U/mL) on proliferation activity of M/A-treated leukemic lymphocytes, analyzed by trypan blue staining and automated cell counting. Figure 3S: correlation analysis between cell proliferation/viability and ROS of menadione/ascorbate-treated cells, described in Figures 2, 4, and 8 (two-sample equal variance). Figure 4S: effect of ferrostatin (Fer-1; 2 μM) and necrostatin (Nec-1; 20 μM) on proliferation activity of M/A-treated leukemic lymphocytes (Jurkat), analyzed by trypan blue staining and automated cell counting. [file 6212935.f1.docx]

**Supplementary materials**

***Appendix 1***

In the present article, we present a full systematic study, which aims to answer the following questions:

1. What is the relative contribution of mitochondrial redox-cycling of ascorbate and menadione to the induction of severe oxidative stress in M/A-treated cancer cells compared to the cytosolic and extracellular redox-cycling mechanism(s) of both substances?
2. Whether M/A induces synergistic overproduction of mitochondrial superoxide in cancer cells or this effect is inherent for menadione and ascorbate applied separately?
3. Is the anticancer effect specific to the combination of M/A or is it inherent to the combinations of ascorbate with other forms of vitamin K (K1, K2), as well as for dehydroascorbate and menadiol?
4. Are there any redox-modulating and immuno-modulating effects of low/tolerable doses of M/A that can sensitize cancer cells to conventional anticancer therapy, as well as to the native immune response?

**
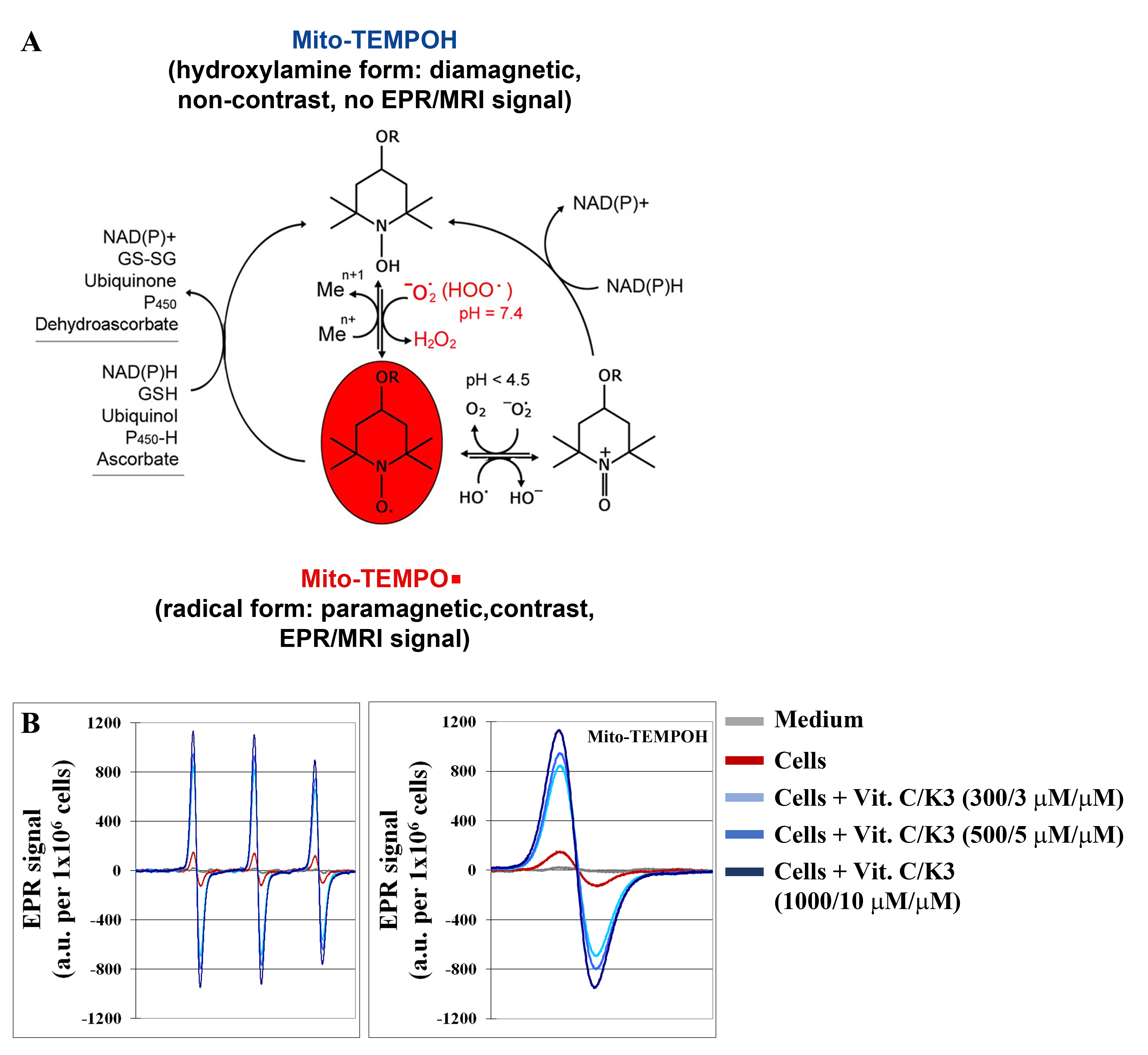
**

**Figure 1S.** (A) Nitroxide redox cycle as a sensing platform for the EPR/MRI imaging of cellular redox-status: principle of the method. In vitro studies show that the nitroxide radical, which is characterized by MRI/EPR contrast enhancement, can be converted to the non-contrast hydroxylamine and/or oxoammonium by different compounds in cells and body fluids (e.g., free ions of transition metals, hydroxyl and hydroperoxyl radicals, ubiquinols, NAD(P)H, ascorbate, glutathione, etc.). The contrast-enhancing radical form can be recovered by interaction of hydroxylamine with the superoxide radical at physiologic pH (7.4) or interaction of oxoammonium with the superoxide radical at pH < 4.5. Thus, the nitroxide-enhanced MRI/EPR signal follows the reduction/oxidation of the nitroxide derivative and indicates the redox-status of cells, tissues and body fluids. (B) Effect of ascorbate/menadione on the EPR signal dynamics of diamagnetic mito-TEMPOH in leukemic lymphocytes – representative EPR spectra from two independent experiments for each sample. Experimental conditions: Cells (1×10^6^ cells/mL) were incubated with ascorbate/menadione for 36-hours in humidified atmosphere. Then, cells were washed and adjusted to 1×10^6^ cells/mL for each sample. Mito-TEMPOH (2 mM) was added to each cell suspension and incubation was continued for 12-hours in humidified atmosphere. EPR spectra were recorded at the parameters, described in Materials and Methods. Negative control – mito-TEMPOH in cultured medium. Positive control – mito-TEMPOH in untreated cells. Both controls are treated in the same way as the samples.

**References:**

1. Bakalova R, Zhelev Z, Aoki I, Saga T. Tissue redox activity as a hallmark of carcinogenesis: from early to terminal stages of cancer. Clin. Cancer Res. 2013, 19(9), 2503-2517.
2. Zhelev Z, Aoki I, Gadjeva V, Nikolova B, Bakalova R, Saga T. Tissue redox activity as a sensing platform for imaging of cancer based on nitroxide redox cycle. Eur. J. Cancer 2013, 49(6), 1467-1478.
3. Zhelev Z, Bakalova R, Aoki I, Lazarova D, Saga T. Imaging of superoxide generation in the dopaminergic area of the brain in Parkinson`s disease, using mito-TEMPO. ACS Chem. Neurosci. 2013, 4(11), 1439-1445.
4. Bakalova R, Georgieva E, Ivanova D, Zhelev Z, Aoki I, Saga T. Magnetic resonance imaging of mitochondrial dysfunction and metabolic activity, accompanied by overproduction of superoxide. ACS Chem. Neurosci. 2015, 6(12), 1922-1929.
5. Georgieva E, Zhelev Z, Aoki I, Bakalova R, Higashi T. Detection of redox imbalance in normal lymphocytes with induced mitochondrial dysfunction – EPR study. Anticancer Res. 2016, 36(10), 5273-5279.
6. Zhelev Z, Georgieva E, Lazarova D, Semkova S, Aoki I, Gulubova M, Higashi T, Bakalova R. “Redox imaging” to distinguish cells with different proliferative indexes: Superoxide, hydroperoxides, and their ratio as potential biomarkers. Oxid. Med. Cell. Longev. 2019, 2019, art. 6373685.

**
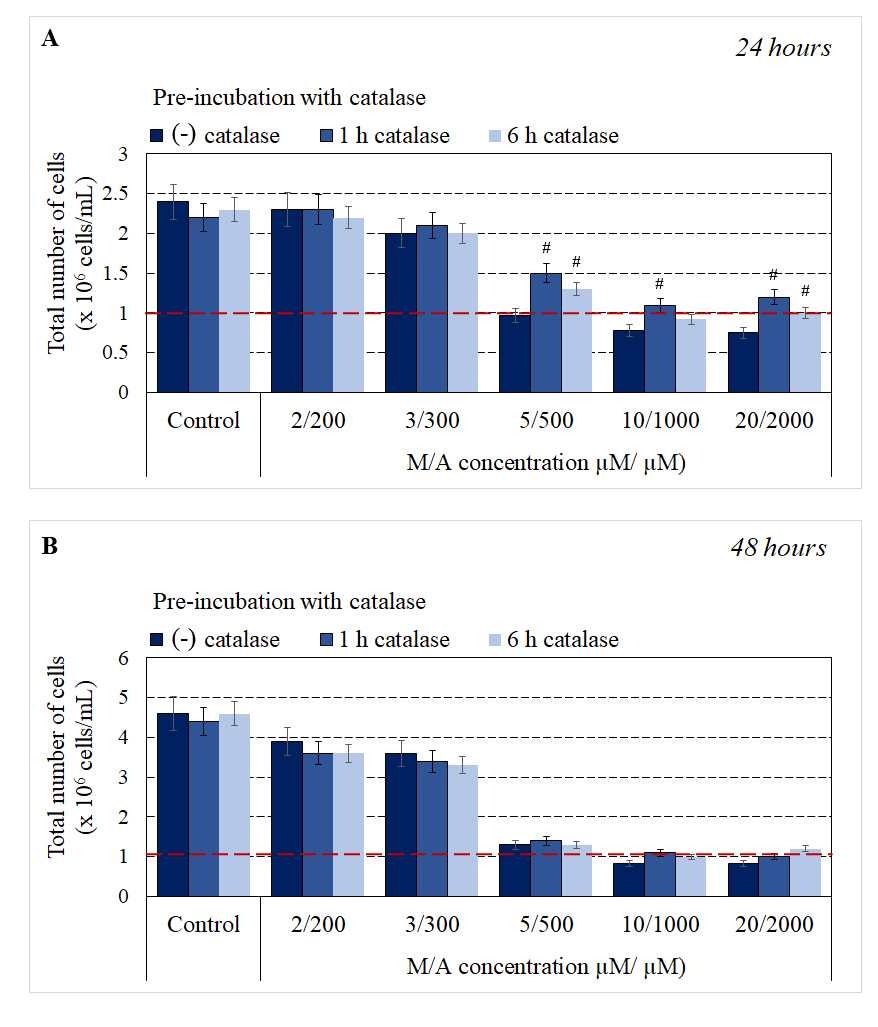
**

**Figure 2S.** Effects of catalase (150 μg/mL or ~500-750 U/mL)* on proliferation activity of M/A-treated leukemic lymphocytes, analyzed by trypan blue staining and automated cell counting. Cells were treated with M/A in the absence and presence of catalase for 1-hour and 6-hours, after that collected by centrifugation (1000xg for 10 min), washed once by PBS, re-suspended in fresh PRMI1640 medium, normalized again to equal cell number in each sample (1×10^6^ cells/mL), and incubation was continued for 24-hours and 48-hours in humidified atmosphere. Controls contained cells treated in the same way, but in the absence of M/A. The red dotted lines indicate the initial number of cells before addition of M/A. The data are means±SD from six independent experiments. #p<0.05 versus the respective catalase (-) sample. The variations between 1-hour and 6-hours treatment with catalase were statistically insignificant.

*In this concentration, catalase decomposed hydrogen peroxide within a minute in cell-free medium with release of oxygen observed even microspopically.

**
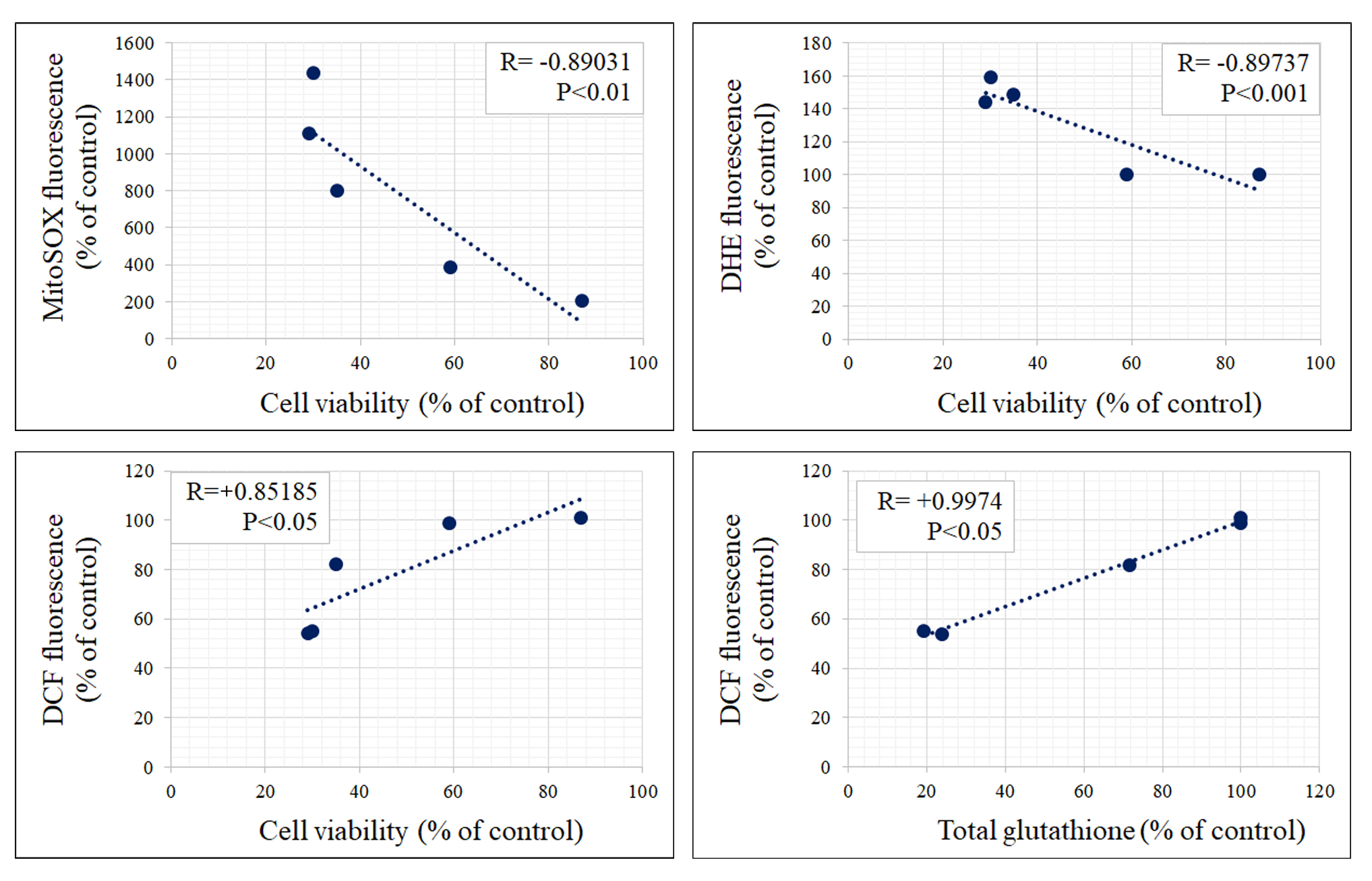
**

**Figure 3S.** Correlation analysis between cell proliferation/viability and ROS of menadione/ascorbate-treated cells, described in Figures 2, 4 and 8 (two-sample equal variance).


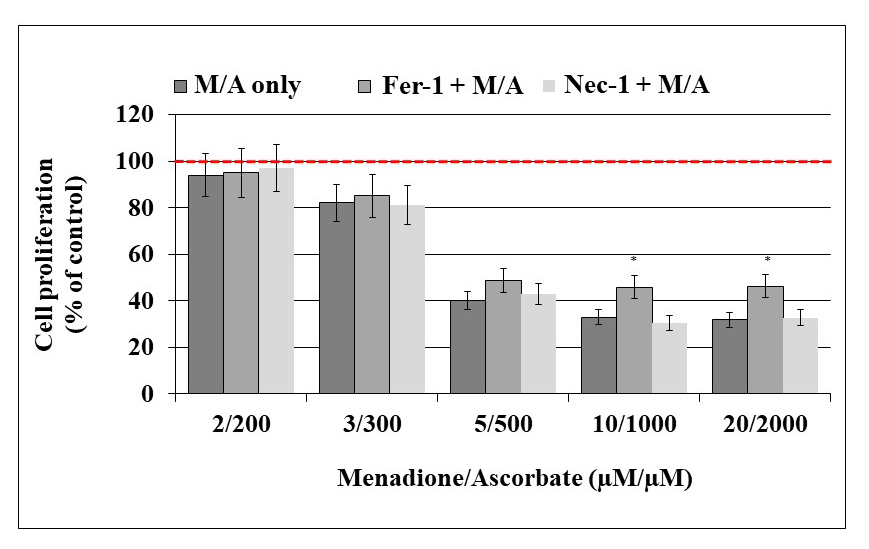


**Figure 4S.** Effect of ferrostatin (Fer-1; 2 μM) and necrostatin (Nec-1; 20 μM) on proliferation activity of M/A-treated leukemic lymphocytes (Jurkat), analyzed by trypan blue staining and automated cell counting. Incubation conditions: Cells were pre-incubated with Fer-1 or Nec-1 for 3-hours, treated with M/A, and incubation was continued for 24-hours in cell incubator. Untreated cells were used as control. The data are presented as a percentage of untreated (control) cells. Fer-1 and Nec-1, applied alone, did not affect significantly cell viability and proliferation of M/A-untreated cells. Means±SD from three independent experiments are shown in the figure.
